# A miniaturized mode-of-action profiling platform enables high throughput characterization of the molecular and cellular dynamics of EZH2 inhibition

**DOI:** 10.1038/s41598-023-50964-x

**Published:** 2024-01-19

**Authors:** Lilia Falkenstern, Victoria Georgi, Stefanie Bunse, Volker Badock, Manfred Husemann, Ulrike Roehn, Timo Stellfeld, Mark Fitzgerald, Steven Ferrara, Detlef Stöckigt, Carlo Stresemann, Ingo V. Hartung, Amaury Fernández-Montalván

**Affiliations:** 1 Bayer AG, Müllerstrasse 178, 13353 Berlin, Germany; 2 Broad Institute, Merkin Building, 415 Main St, Cambridge, MA 02142 USA; 3 Present Address: Rentschler Biopharma SE, Erwin-Rentschler-Straße 21, 88471 Laupheim, Germany; 4 Present Address: Nuvisan Innovation Campus Berlin, Müllerstrasse 178, 13353 Berlin, Germany; 5 Present Address: Nested Therapeutics, 1030 Massachusetts Avenue, Suite 410, Cambridge, MA 02138 USA; 6 Present Address: Merck KGaA, Frankfurter Str. 250, 64293 Darmstadt, Germany; 7 Present Address: Boehringer Ingelheim Pharma GmbH & Co. KG, Birkendorfer Str. 65, 88400 Biberach an der Riß, Germany

**Keywords:** High-throughput screening, Mechanism of action, Biochemistry, Biophysics

## Abstract

The market approval of Tazemetostat (TAZVERIK) for the treatment of follicular lymphoma and epithelioid sarcoma has established “enhancer of zeste homolog 2” (EZH2) as therapeutic target in oncology. Despite their structural similarities and common mode of inhibition, Tazemetostat and other EZH2 inhibitors display differentiated pharmacological profiles based on their target residence time. Here we established high throughput screening methods based on time-resolved fluorescence energy transfer, scintillation proximity and high content analysis microscopy to quantify the biochemical and cellular binding of a chemically diverse collection of EZH2 inhibitors. These assays allowed to further characterize the interplay between EZH2 allosteric modulation by methylated histone tails (H3K27me3) and inhibitor binding, and to evaluate the impact of EZH2’s clinically relevant mutant Y641N on drug target residence times. While all compounds in this study exhibited slower off-rates, those with clinical candidate status display significantly slower target residence times in wild type EZH2 and disease-related mutants. These inhibitors interact in a more entropy-driven fashion and show the most persistent effects in cellular washout and antiproliferative efficacy experiments. Our work provides mechanistic insights for the largest cohort of EZH2 inhibitors reported to date, demonstrating that—among several other binding parameters—target residence time is the best predictor of cellular efficacy.

## Introduction

Enhancer of zeste homolog 2 (EZH2), the enzymatic subunit of the Polycomb repressive complex 2 (PRC2) has recently joined the list of clinically validated oncology drug targets with the FDA approval of the small molecule inhibitor tazemetostat (TAZVERIK) for the treatment of advanced epithelioid sarcoma^1–3^ and follicular lymphoma^4^. PRC2 is one of two PcG protein core complexes encoded by the polycomb (PcGs) group genes that regulate transcription via post-translational mono-, di- or trimethylation of Lys-27 in histone 3 (H3K27me3)^5,6^ by EZH2. From these modifications, H3K27me1 is found in active genes, whereas H3K27me2 and H3K27me3 are associated with global repression of gene expression, and thus considered to be a key epigenetic hallmark during tissue development and stem cell fate determination^6^. In addition, PRC2 has been reported to trigger target gene activation via chromatin-independent mechanisms^7^. Owing to the above-described roles, PRC2 acts non-redundantly to PRC1 (the second PcG complex, with distinct composition and biochemical mechanism^8,9^) at target genes to maintain transcriptional programs and ensure cellular identity^10,11^. In a pathological context, PRC2 is involved in cancer initiation, progression, metastasis, metabolism, drug resistance, and immunity regulation. For these reasons PRC2’s catalytic subunit EZH2 was considered in the first place as an attractive drug target for cancer therapy^12,13^.

EZH2 catalyzes the transfer of a methyl group from the cofactor S-adenosyl methionine (SAM) to the lysine-27 residue of histone 3 via its C-terminal SET-domain that belongs to the class of histone lysine methyltransferases (HMTs). In some complexes EZH2 is replaced by its paralog EZH1, a substitution that confers distinct biological functions to PRC2, despite its high homology (94%). Besides the SET-domain, four additional subunits (EED, SUZ12, RbAp48 and AEBP2) complete the composition of this multimeric protein to an estimated molecular mass of 275 kDa. Among them, embryonic ectoderm development (EED) and suppressor of zeste 12 (SUZ12) directly contact EZH2 and are essential for methyltransferase activity of EZH2^8,14–19^.

Disease-related heterozygous point mutations within the SET-domain of EZH2 mainly found in lymphomas and myeloid leukemias, alter the substrate specificity of EZH2 to generate different methylation states of H3K27 which promote oncogenic progression. While wild-type EZH2 specifically uses unmethylated H3K27, the disease-associated EZH2 Y641N mutant is completely unable to catalyze the first methylation reaction of H3K27 but exhibits increased activity for di- and trimethylation reactions^20–23^.

Besides modulating chromatin structure and transcriptional status, the H3K27me3 modification is implicated in a regulatory feedback loop, which involves binding to the EED subunit of PRC2 and concomitant conformational changes in the SET domain of EZH2 that result in higher affinity for the SAM co-substrate and increased catalytic efficiency^24,25^. This role of H3K27me3 as stimulator of EZH2 activity makes the EED binding site a target for allosteric inhibitors^26–29^. In addition, the H3K27me3 histone mark can modulate the binding properties of active site inhibitors^30^.

Tazemetostat and most EZH2 drug candidates in pre-clinical and clinical development are orthosteric small molecule inhibitors. They share a common pyridone motif, which interacts with the backbone amide of the W624 residue via hydrogen bond interactions of its N–H and C=O groups. EZH2 SET domain’s W624 residue establishes a critical H-bond to the terminal amino acid moiety of the SAH/SAM cofactor during catalysis, and this interaction is replaced by the pyridone ring in the inhibited state, which explains the competitive mode of action of these compounds. Intriguingly, the search for chemical alternatives to the pyridone motif has not been successful so far, but combinations of this scaffold with a variety of other rings (e.g. indazole, indole or aniline) has generated EZH2 inhibitors with diverse physicochemical and pharmacological profiles (see Martin et al.^31^ for a comprehensive overview).

One differentiating factor of current EZH2 drug candidates is their target residence time^30,32,33^, an increasingly recognized optimization parameter in drug discovery which can influence the safety, pharmacokinetics (PK), and pharmacodynamics (PD) of therapeutic agents^34–37^. Preliminary studies of GSK’s indole series suggested that target occupancy can be prolonged by allosteric binding of H3K27me3, resulting in increased cellular potency^30^. More recently, optimization of second generation EZH2 inhibitors was guided by binding kinetics information generated in the absence and presence of H3K27me3^32,33^. These few examples are of unique value, given the technical complexity of conducting ligand binding energetics and dynamics studies with a complex multimeric complex such as PRC2. Thus—despite the progress made—our current knowledge of EZH2’s molecular pharmacology is still limited to small datasets covering representatives from a few compound classes.

With these challenges in mind and aiming to support our own EZH2 drug discovery efforts^38^, we set out to develop high throughput assays for large-scale assessment of the kinetics and thermodynamics parameters of EZH2 inhibitor interactions in a cell-free context, as well as in in vitro cellular models. All assays were validated with the largest and more structurally diverse cohort of known EZH2 inhibitors studied so far. Our results provide new mechanistic insights for these compounds and constitute a basis to future non-equilibrium quantitative systems pharmacology (QSP) and PK/PD modelling studies for EZH2 drug candidates.

## Results

### Development of TR-FRET based equilibrium and kinetic binding competition assays for the characterization of EZH2 inhibitors

**Figure 1:**
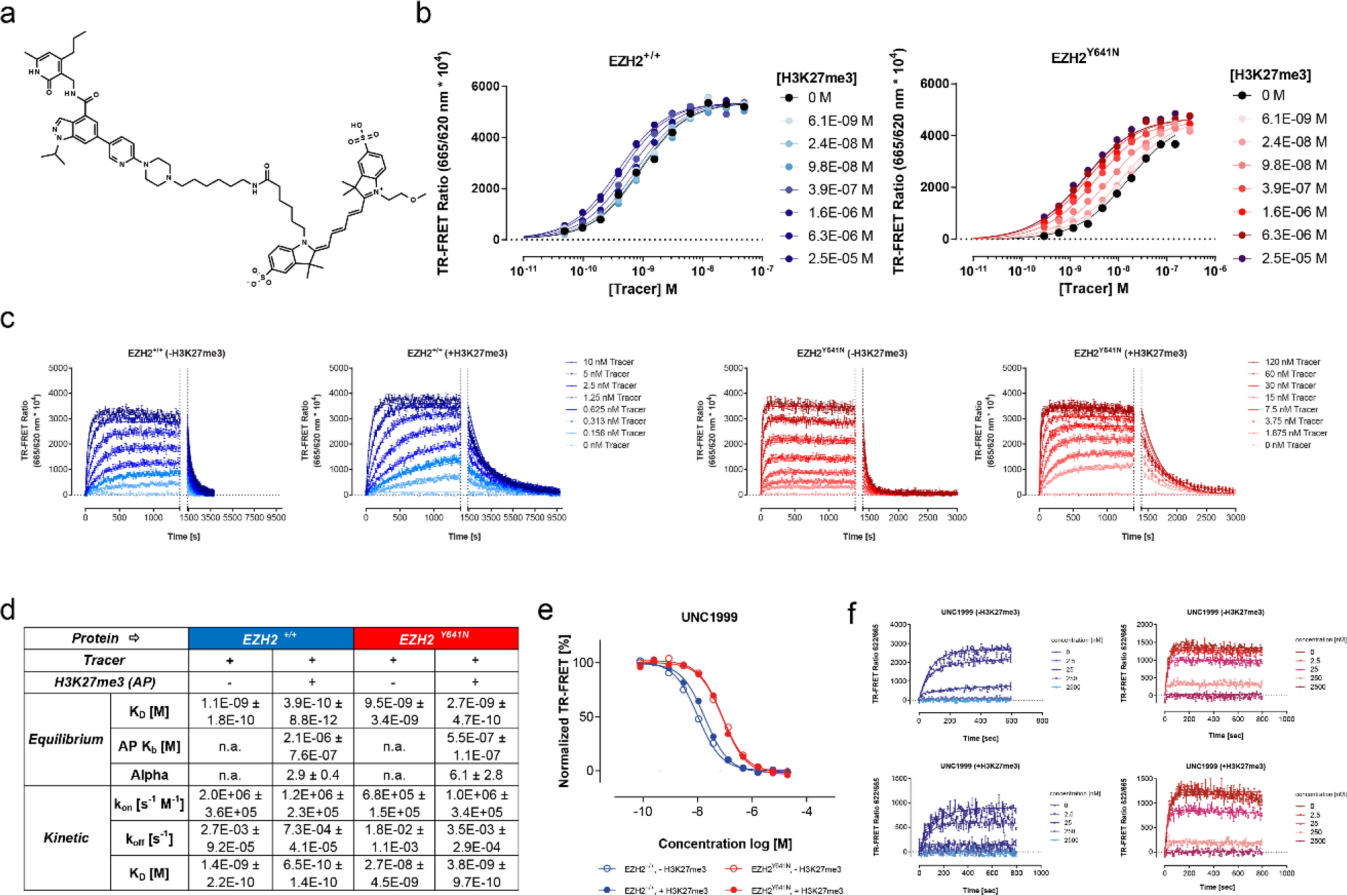
Development of a fluorescent probe and homogeneous TR-FRET competition assays for the characterization of EZH2 inhibitors. (**a**) Chemical structure of the fluorescent probe synthesized for this study. (**b**) Equilibrium titrations of the probe on wild type (^+/+^) and mutant (^Y641N^) EZH2 performed in presence of increasing concentrations of H3K27me3 allosteric activator peptide (indicated on the right-hand side of the graphs). Fitting these curves to 1:1 single site- and allosteric binding models allowed calculating the K_D_ and K_b_ values shown in panel d and the Supplementary Table S1. Here and along the entire paper, data for EZH2^+/+^ and EZH2^Y641N^ are represented by blue and red dots and lines, respectively. (**c**) Kinetic titrations of the probe on EZH2^+/+^ and EZH2^Y641N^ in the absence (left panel) or presence (right panel) of saturating concentrations of H3K27me3 activator peptide. Increasing concentrations of the probe (indicated on the right-hand side of each pair of graphs) were mixed with labeled enzyme and TR-FRET signals were recorded for 4 min. Equilibrium complexes were then disrupted by the addition of an excess amount of the unlabeled compound (marked with dotted vertical lines in the graph) and dissociation of the probe was followed until baseline fluorescence ratios were reached. Fitting of these kinetic traces to a global association and dissociation model led to the probe rate- and affinity constants shown in panel d, and the Supplementary Table S1. (**d**) Equilibrium and kinetic binding parameters of the fluorescent probe on EZH2^+/+^ and EZH2^Y641N^ in the absence or presence of H3K27me3 activator peptide. (**e**) Exemplary normalized equilibrium curves of fluorescent probe displacement from wild type and mutant EZH2 by reference inhibitor UNC1999. Open and filled circles show competition in the absence and presence of saturating concentrations of the H3K27me3 peptide. Fitting these curves to a 4-parameter logistic model (solid lines), and conversion of the resulting IC_50_ values to Ki values with the Cheng-Prusoff equation, led to affinity parameters shown in the Supplementary Spreadsheet. (**f**) Representative kinetic probe competition assay (kPCA) traces for the same exemplary compound in the absence and presence of saturating concentrations of the H3K27me3 peptide. Fitting these curves to the Motulsky and Mahan model for competitive binding kinetics (solid lines) yielded the kinetic parameters shown in the Supplementary Spreadsheet.

To pursue our objectives, we followed methods previously established in our laboratory to develop equilibrium- and kinetic probe competition assays with homogeneous time resolved fluorescence energy transfer (TR-FRET) readout (ePCA and kPCA, respectively)^39,40^. To this end, we synthesized a fluorescent probe molecule derived from UNC2239^41^ containing an Alexa 647 dye instead of Cy5 for improved TR-FRET acceptor properties (Fig. 1a). Concomitantly, we generated chemically biotinylated EZH2 wild type and Y641N proteins to enable tight labeling with streptavidin-coupled TR-FRET donors. Mass spectrometry analysis of the reaction products showed that this modification did not compromise critical active site residues (Fig. S1).

With these tools in hands, we conducted cross titrations of PRC2 complexes versus the fluorescent probe (data not shown) to determine the equilibrium affinity of the interactions (Fig. 1d and Supplementary Table S1) and the smallest amounts of binding partners required to obtain sufficiently robust assay signals. In the next step, protein concentrations were fixed at the lowest possible levels to avoid ligand depletion, and probe titrations were repeated in the absence and presence of increasing amounts of H3K27me3 peptide (Fig. 1b). In these experiments we observed small but significant dose-dependent effects of the activator peptide on the probe binding saturation curves, allowing to perform global fitting of these curves to an allosteric affinity shift model^42^. This analysis helped determine both the probe’s EC_50_ and the H3K27me3 peptide affinities (K_b_) for its allosteric binding site as well as the ternary complex constant (alpha) (Fig. 1d and Supplementary Table S1).

The affinity values of 1.1 ± 0.2 and 0.4 ± 0.01 nM in equilibrium conditions obtained for the fluorescent ligand and wild type EZH2 (EZH2^+/+^) in the absence and in the presence of saturating concentrations of H3K27me3 peptide are aligned with the Ki values of 2.8 ± 0.9 and 1.1 ± 0.5 nM measured for the parental compound UNC1999 (Fig. 1e and “9” in the Supplementary Spreadsheet), suggesting that the Alexa647 moiety does not contribute significantly to the binding energy. However, these affinities are significantly higher than the IC_50_ values of 10 nM and 20 nM reported for UNC1999 and UNC2239, respectively, by Konze et al., supporting the authors’ postulate that their potency values were bottomed out due to the 40-fold higher enzyme concentration used^41^. Further confidence on our assay was provided by the fact that H3K27me3 peptide titration stimulated probe binding to EZH2^+/+^ with a K_b_ of 2.1 ± 0.8 µM, a value which is in excellent agreement with the K_D_ value of 3.3 ± 0.4 µM obtained in the presence of SAM by Justin et al.^17^ in fluorescence polarization competition experiments. Likewise—as expected from previous evidence on the cross talk between EZH2’s catalytic and regulatory subunits^17,24,43^—the Y641N mutation results in a fourfold decreased K_b_ of 0.5 ± 0.1 µM for the allosteric H3K27me3 peptide.

Next, we characterized the binding kinetics of our fluorescent probe in the absence and presence of saturating concentrations of the H3K27me3 peptide. Figure 1c shows typical curves corresponding to fluorescent probe association and dissociation obtained in both conditions, and their fit to the 1:1 kinetic binding model derived from the law of mass action. The parameters derived from this analysis demonstrate that both allosteric modulation and the Y641N mutation influence the probe’s rate constants (Fig. 1d and Supplementary Table S1). More precisely, binding of the H3K27me3 peptide to EZH2^+/+^ results in 27-fold slower dissociation rates, without noticeable impact on the association rates. Likewise, the Y641N mutation leads to 19-fold slower off-rates (−/+ allosteric activation) while the on-rates did not change significantly.

Having assessed binding parameters for both the fluorescent probe and the H3K27me3 peptide in a variety of conditions, we went on to determine the appropriate concentrations of probe, allosteric modulator, and proteins to conduct equilibrium and kinetic binding competition assays. These protocols were validated with literature compounds^31^ such as Tazemetostat (EPZ-6438, or “2” for free base and “3” for salt form in this study), GSK126 (“4”), UNC1999 (“9”), CPI-169 (“10”), Pfizer Cmpd. 31 (“14”) and DS-3201 (“31”) (Fig. S2). Representative ePCA and kPCA curves obtained for some of these inhibitors in the absence and presence of the H3K27me3 activator peptide, fits to the corresponding binding models, and the binding parameters derived from these fits are displayed in Fig. 1e–f, Fig. S3 and the Supplementary Spreadsheet.

The equilibrium and kinetic affinity values obtained from both assays were highly correlated, and in fair agreement with the IC_50_ for EZH2 inhibition (Fig. S4 and Supplementary Spreadsheet). More importantly, considering the differences in assay formats and conditions used, both the ranking and the absolute residence times of GSK126, EPZ-6438 and DS-3201 were comparable to the values reported in previous studies^30,33^. Only for the slowest interactions in the dataset (especially those in presence of H3K27me3), our standard kPCA conditions did not allow for accurate k_off_ determinations with the competitive binding kinetics model. Based on our previous in-depth investigation of this phenomenon^44^, and for the sake of homogenous comparison between all compounds, we employed the recommended procedure of calculating dissociation constants by multiplying the k_on_ and the equilibrium K_D_ values^39,44^. It should however be noted that these affinities in equilibrium conditions might be influenced by ligand depletion and elusive equilibrium^45^ effects, and some calculated off-rate values (including those for reference compounds 2, 3, 10 and 31) could have been underestimated.

### Mechanistic characterization and structure-kinetic relationship (SKR) of orthosteric EZH2 inhibition

**Figure 2:**
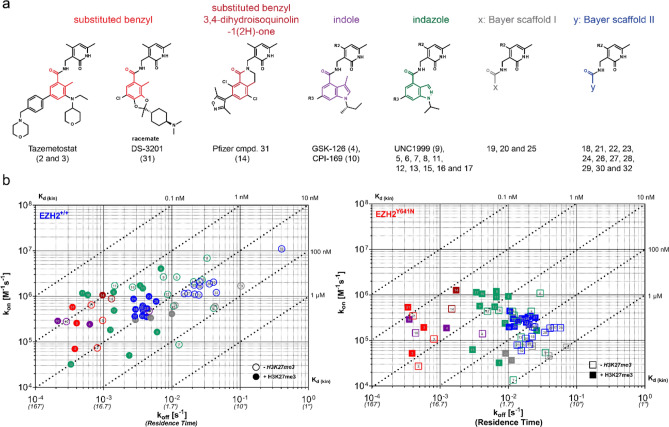
Kinetics of EZH2 binding by pyridone-based active site inhibitors. (**a**) Structural features of the EZH2 inhibitor set investigated in this study. All compounds share a common pyridone motif in the north side of the molecule and are differentiated by distinct chemical cores highlighted in different colors. For two of the Bayer-proprietary chemical series, distinctive colors were only applied to label the linkage between the pyridone and the undisclosed heterocyclic scaffolds. Whenever relevant (and feasible), this color code was subsequently used to label compounds throughout the entire paper. (**b**) Rate plots with isoaffinity diagonals (RAPID) representing the binding to wild type and mutant EZH2 of all compounds investigated in this study. Compounds are colored and numbered as in panel a for visualization of structure kinetic relationships (SKR). Open or filled symbols represent data generated in the absence or presence of H3K27me3 peptide.

Following validation with reference inhibitors, we evaluated the applicability of this new ePCA and kPCA methods for high throughput profiling of structure-kinetic relationships (SKR) in our EZH2 drug discovery program. Here we show data for 32 compounds, including best known Epizyme and Daichi’s substituted benzyls, Pfizer’s published dihydroisoquinolinone variation thereof, GSK’s and Constellation’s indoles, as well as the indazoles previously disclosed by the Structural Genomics Consortium (SGC) and Bayer^38^. In addition, we tested compounds from two undisclosed Bayer-proprietary series, and an inactive control (1) whose corresponding negative results were omitted from the manuscript. Altogether the set of inhibitors chosen for this study represents six different chemical scaffolds sharing a common pyridone motif present in many EZH2 inhibitors reported so far. These compounds are decorated with various substitution patterns around their core motifs, a useful feature to enable the study and understanding of structure-kinetics relationships (Fig. 2a).

The binding kinetics parameters obtained for these compounds in the absence and presence of activator peptide can be found in the Supplementary Spreadsheet. These data allowed representation of general features of their interactions with EZH2^+/+^ and EZH2^Y641N^ in rate plots with isoaffinity diagonals (RaPID) (Fig. 2b), and to quantify the effects on the individual affinity and rate constants of the mutation and the H3K27me3 peptide (Fig. S5). Visual inspection of the RaPID plots shows compound affinities spreading across two and three orders of magnitude for the wild type and mutant EZH2, respectively. As previously described in the literature, the Y641N mutation has a (≥ tenfold) negative impact on inhibitor binding. This was especially true for the indazoles and the Bayer proprietary scaffolds. On the other hand, the presence of the H3K27me3 peptide resulted in a small but consistent increase in affinity ranging between 0.5 and 1 log.

As for the individual rate constants, the on-rates only changed nearly three orders of magnitude (*ca.* 10^4^–10^7^ for EZH2^+/+^ and *ca.* 10^3^–10^6^ M^−1^ s^−1^ for EZH2^Y641N^) between the slowest and the fastest associating compounds, whereas the off-rates were distributed across a wider range, with residence times varying from a few seconds to nearly 1 h or longer (see comments above on the accuracy of k_off_ determinations). The analysis in Fig. S5 shows that adding approximately threefold excess amounts of the H3K27me3 peptide relative to its K_b_ (10 µM for EZH2^+/+^ and 1.5 µM for EZH^Y641N^) resulted in slower dissociation rates, with log differences ranging between 0.5 and 1. However, the magnitude of these changes is rather compound-specific—a result to be considered when analyzing correlations between potencies from inhibition assays and affinities from binding experiments. This visualization also suggest that the Y641N mutation mainly impairs association by small molecules, since k_on_ is the most affected parameter, especially in the absence of H3K27me3 peptide. Based on the magnitude of changes in both rate constants, target residence time appears to be the most actionable affinity optimization handle for orthosteric EZH2 inhibitors.

From a structure-kinetic relationships (SKR) perspective, a few trends are evident in the RaPID visualization presented in Fig. 2b: first, the indoles (purple) and substituted benzyls (red) displayed the slowest dissociation rates, along with some members from the indazole (green) series, which typically showed the fastest association rates. Not surprisingly, these chemotypes exhibited the highest affinities for the two EZH2 variants, nevertheless the affinity shift between the wild type and mutant EZH2 was clearly smaller for the substituted benzyls (Fig. S5). Interestingly, the salt form of EPZ-6438 (3) displays a significantly higher on-rate as compared to its free base (2), pointing to the impact that increased solubility may have on the ligand concentration component of this second order parameter. Finally, none of the Bayer proprietary scaffolds displayed affinities and residence times comparable to the published inhibitors and none of the substituents inspired from more advanced compounds of the other classes seemed to significantly ameliorate their binding parameters.

### Equilibrium and transition-state thermodynamics of orthosteric EZH2 inhibitor binding

**Figure 3:**
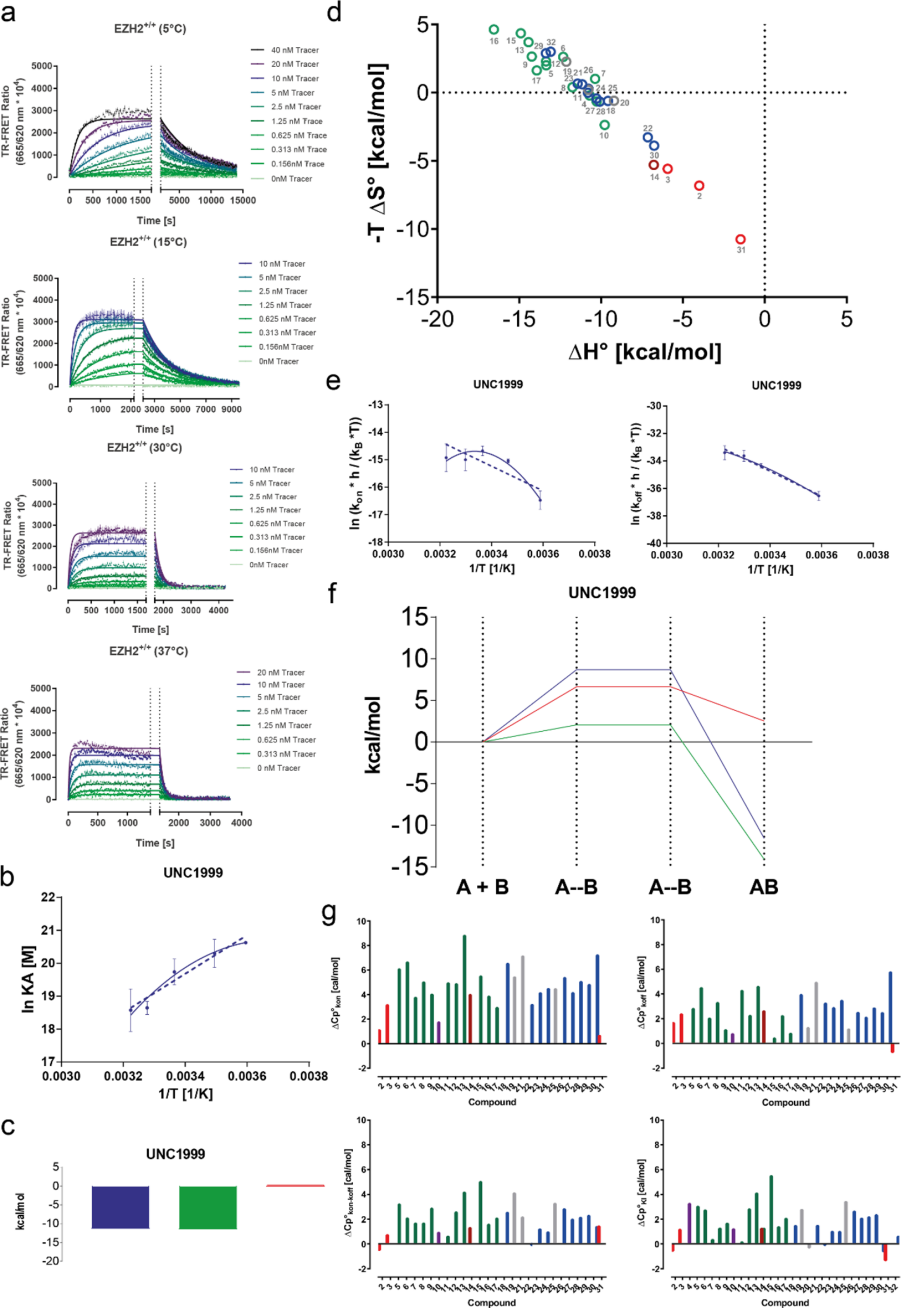
Equilibrium and transition state thermodynamics of EZH2 binding by pyridone-based active site inhibitors. (**a**) Kinetic titrations of the probe on EZH2^+/+^ at different temperatures (indicated on the label of each graph). Increasing concentrations of the probe (provided on the right-hand side of each graph) were mixed with labeled enzyme and TR-FRET signals were recorded for 4 min. Equilibrium complexes were then disrupted by the addition of an excess amount of the unlabeled compound (marked with dotted vertical lines in the graph) and dissociation of the probe was followed until baseline fluorescence ratios were reached. Fitting of these kinetic traces to a global association and dissociation model led to the probe rate- and affinity constants shown in the Supplementary Table S1. (**b**) Equilibrium thermodynamics analysis derived from ePCA experiments for exemplary reference EZH2 inhibitor UNC1999. Dotted lines represent the fit of the data to the linear form of the van’t Hoff equation, whereas solid lines show the fit to the polynomial one. (**c**) Representative equilibrium thermodynamics profile of reference EZH2 inhibitor UNC1999. The blue, green, and red bars represent the Gibbs’ free energy (ΔG) enthalpy (ΔH), and entropy (-TΔS) components of the binding, respectively. (**d**) Equilibrium thermodynamic optimization plots of the 32 compounds investigated in this study. The labeling and colors of the data points describe compound names and structures as indicated in Fig. 2a and the Supplementary Spreadsheet. (**e**) Transition state thermodynamics analysis derived from kPCA experiments for exemplary reference EZH2 inhibitor UNC1999. Dotted lines represent the fit of the data to the linear form of the Eyring equation, whereas solid lines show the fit to the polynomial one. The left graph shows the analysis for the association phase, whereas the right graph displays the dissociation phase. (**f**) Transition state thermodynamics profile of exemplary reference EZH2 inhibitor UNC1999 investigated in this study. The blue, green and red bars represent the Gibbs’ free energy (ΔG) enthalpy (ΔH), and entropy (-TΔS) components of the binding, respectively. (**g**) Overview of heat capacity changes (ΔCp) for each compound investigated in this study. The upper panel shows kinetic ΔCp values derived from kPCA in the association (left hand side) and dissociation (right hand side) phases. The lower panel shows equilibrium ΔCp values derived from kPCA (left-hand side) and ePCA (right-hand side). The labeling and colors of the data points describe compound names and structures as indicated in Fig. 2a and the Supplementary Spreadsheet.

Protein availability issues and insufficient compound solubility had prevented us from using isothermal calorimetry (ITC) at a reasonably large scale for the profiling of EZH2 inhibitors during the lead finding and optimization phases. These challenges motivated us to ask whether our ePCA and kPCA protocols could be used to generate binding data at a wider range of temperatures for subsequent van’t Hoff and Eyring analysis. To this end (using wild type EZH2 as target protein) we further extended the binding kinetics characterization of the fluorescent probe (previously performed at 25 °C) to 5, 15, 30 and 37 °C (Fig. 3a). The traces obtained from these experiments contained sufficient kinetic information to determine the rate constants of the fluorescent probe at each temperature tested: as expected, the rates of probe’s association and dissociation kinetics were temperature-dependent, with a clear trend towards faster binding with increasing temperatures (Supplementary Table S1).

The results described above encouraged us to perform equilibrium and kinetic competition assays at these temperatures for all compounds investigated (data not shown). The ePCA curves obtained under these conditions allowed to extract the affinities from which binding enthalpies and entropies were calculated using both the linear^46^ and polynomial^47^ van’t Hoff equations (Supplementary Spreadsheet). Figures 3b and S6a show the fits of these equations for the reference inhibitors and the equilibrium thermodynamic signatures obtained for these compounds (Figs. 3c and S6b). Based on these data, most interactions are enthalpy-driven, except for reference compounds 2, 3, 4, 14 and 31, for which the polynomial van’t Hoff analysis predicts entropy-driven binding. Among them, only the clinical stage inhibitors Tazemetostat and DS-3201 are entropy-driven binders in the linear model, a consistent pattern that inspired us to represent these data in a thermodynamic optimization diagram^48^, using the parameters obtained from the non-linear van’t Hoff analysis (Fig. 3d). As previously shown for other drug classes such as the statins and HIV protease inhibitors^49^, only the development candidates mentioned above and other advanced inhibitors enter or approach the “entropy-dominance” zone of the graph^50^, supporting the usefulness of these plots to identify compounds that can potentially overcome the hurdles of pre-clinical development.

The van’t Hoff plots for EPZ-6438 and GSK126 (Fig. S6a) represent the two differentiated profiles that we observed across the dataset: one where both the linear and polynomial models deliver similar results (exemplified by EPZ-6438) and another one where the polynomial equation provides a better fit to the experimental data (represented by GSK126), suggesting that changes in EZH2 protein’s heat capacity (ΔCp) are not constant for all ligand interactions. This observation motivated us to track the ΔCp of each binding event in addition to the traditional ΔH and -TΔS parameters (Fig. 3g). Here we could not link any thermodynamic pattern to individual chemical classes within the set analyzed in equilibrium conditions. However, the clinical compound Tazemetostat and DS-3201 are the ones displaying the smallest ΔCp values.

The kinetic binding thermodynamics experiments performed at different temperatures delivered kPCA traces, many of which contained sufficient information for deriving on- and off-rates with the Motulsky-Mahan model. This allowed to compute the transition state thermodynamic parameters using both the linear and nonlinear versions of the Eyring equation and generate transition state for most of the compounds analyzed. Eyring analyses for the reference compounds are shown in Figs. 3e–f and S7a, whereas Fig. S7b shows the transition state thermodynamic signatures for all inhibitors characterized in this study. Here again, the shape of the Eyring plots and the overlap between the experimental data points and corresponding fits to the linear and non-linear models was compound specific. Generally, the curvature of the plots for the complex formation phase was greater than for complex dissociation, as indicated by the ΔCp values shown in Fig. 3g.

The transition state thermodynamic signatures derived from these analyses were complex and varied significantly between models, especially for the entropy component, followed by the enthalpy. Here we found the comparison of the ΔCp values as a more straightforward way to visualize differences between individual compounds. As mentioned above, the ΔCp for the complex formation was significantly greater than for dissociation. Intriguingly, there appears to be an inverse correlation between ΔCp k_on_ and the overall potency and affinity of the compounds. Given the roles adjudicated to ΔCp k_on_ values in the context of enzyme conformation during catalysis^51,52^ it is tempting to speculate that EZH2’s most advanced compounds tend to be better at selecting a specific conformational state of the PRC2 complex.

### Impact of compound binding kinetics on biochemical EZH2 inhibition

**Figure 4:**
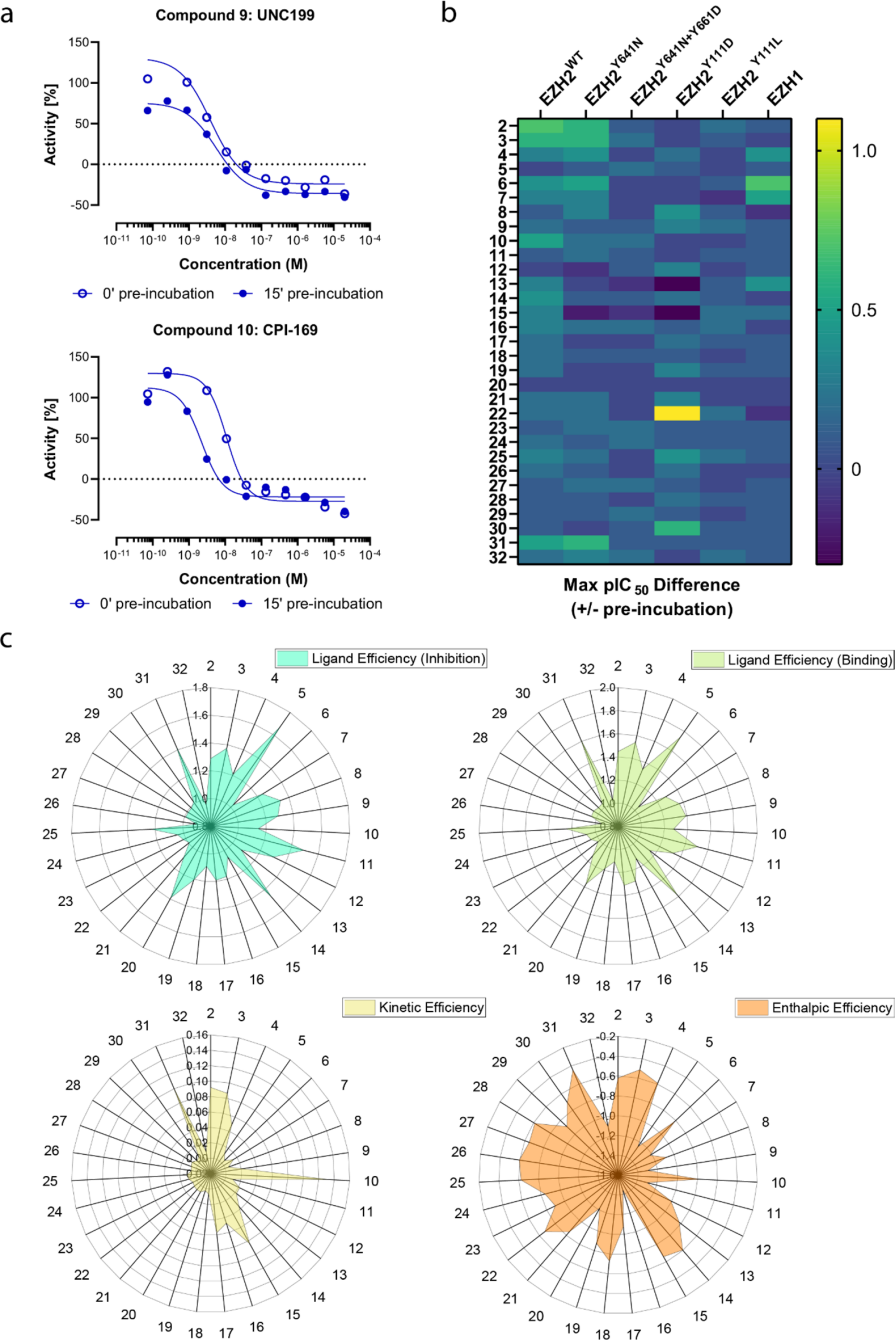
Impact of compound binding kinetics on biochemical EZH2 inhibition and comparison of kinetic-, thermodynamic- and conventional ligand efficiency parameters. (**a**) Representative enzyme inhibition curves for EZH2^+/+^ by two reference inhibitors +/− compound and protein pre-incubation. (**b**) Effects of increasing compound and protein pre-incubation times on the inhibition of different PRC complexes by the compounds analyzed in this study. The color code represents maximum differences in pIC_50_ values +/− compound and protein pre-incubation, using the no pre-incubation pIC_50_ value as reference. The PRC complexes evaluated are indicated on the top. (**c**) Radar plots representing the ligand efficiencies of the compounds analyzed in this study calculated based on their biochemical inhibition (top left), TR-FRET equilibrium binding affinities at 25 °C (top right), residence times derived from TR-FRET kinetic binding (kPCA) assays at 25 °C (bottom left) and enthalpies from TR-FRET equilibrium binding affinities at multiple temperatures (bottom right).

Protracted target residence time due to slow off-rates or high compound rebinding typically results in more effective enzyme inhibition and longer lasting pharmacological effects^36^. In the case of EZH2, two laboratories have shown that biochemical and cellular potency are correlated with slow dissociation rates^30,32,33^. These authors also demonstrated that compound potency was substantially increased in experiments where inhibitor, enzyme and activator peptide were pre-incubated prior to starting the enzyme reactions. We asked whether these observations were applicable to a broader spectrum of chemotypes and determined their potencies  −/+ pre-incubation for comparison with the on- and off-rates measured with kPCA (Supplementary Spreadsheet). The results from these experiments are summarized in Fig. 4. Exemplary inhibition curves of EZH2^+/+^ by UNC199 and CPI-169 with and without 15 min enzyme:compound pre-incubation with wild type shown in Fig. 4a demonstrate that the corresponding shifts in activity are correlated to the residence times.  For a reference compound such as UNC199 the potency shift was very small compared to another one (such as CPI-169) with a noticeable decrease in IC_50_ upon 15 min enzyme:compound pre-incubation. Based on these results, we increased the pre-incubation time up to 1 h and calculated the maximum pIC_50_ difference compared to no pre-incubation for each compound, this parameter was then visualized as heatmap to enable the detection of compounds with significant differences in activity depending on pre-incubation time (Fig. 4b). The compounds’ maximum pIC_50_ differences −/+ pre-incubation were also compared to their residence times (data not shown), and we observed that compounds with residence times beyond 30 min are likely to double their potency upon pre-incubation. Interestingly, a trend towards better correlation between potency shifts −/+ pre-incubation was obtained when k_off_ values were determined in the presence of H3K27me3 peptide (data not shown).

In the next step we expanded the analysis to the clinically relevant single mutants Y641N, Y111D, Y111L and the double mutant Y641N/Y661D^53,54^, as well as to the EZH1 isoform. Figure 4b and the Supplementary Spreadsheet show the inhibition profiles at two to three pre-incubation conditions. Generally, the potency against these protein variants is lower, with the double mutation Y641N/Y661D appearing as the more deleterious, closely followed by Y111D, and the Y111L mutation being the less impactful. Our results confirm the IC_50_ values for inhibition of EZH2^Y641N/Y661D^ reported by Gibaja et al. explaining why the Y111D mutation on the WT allele conferred the most robust resistance to EPZ-6438 and GSK126 treatment in viability assays^53^. Interestingly, the results for DS-3201 illustrate (at least for the substituted benzyls) that chemical optimization can lead to improved compounds with less pronounced acquired resistance issues, especially if combined with a higher activity towards EZH1.

In this dataset we observed a trend towards stronger potency with longer contact times for most compounds and protein variants analyzed. However, the inhibitory activities −/+ compound and enzyme pre-incubation were difficult to quantify for the Y641N/Y661D and Y111L mutants (Supplementary Spreadsheet). The magnitude of these IC_50_ changes correlated with the length of contact time, but—intriguingly—for some inhibitors the potency shift after 15 min pre-incubation was greater than after 1 h. In summary, our data suggest that inhibition assays can be used for rapid qualitative screening of slow binding inhibitors in PRC2 drug discovery programs.

### Comparison of kinetic- and thermodynamic efficiencies with conventional ligand efficiency parameters

Ligand efficiency metrics have gained popularity among drug discovery practitioners as means to normalize compounds affinities and/or functional activities to their molecular size^55–57^. The utility of the classical efficiency metrics has been questioned more than once^58,59^, and in this context, kinetic- and enthalpic efficiencies have been proposed as better predictors of a compound’s potential to progress along the value generation chain^60,61^. With the binding- and inhibition dataset described above, we inquired if and how these metrics could add further value to decision-making in the optimization of orthostheric EZH2 inhibitors.

Figure 4c (top panels) illustrates that the ligand efficiency profiles of the compounds analyzed were similar irrespectively of whether they were calculated based on enzyme inhibition or enzyme binding measurements. While these results were not surprising based on the good correlation between binding and inhibition (Fig. S4), the differences seen for the compounds analyzed were rather moderate. In contrast, the kinetic- and enthalpic efficiency profiles (Fig. 4c, bottom panels) were sharper for the literature compounds benchmarked, suggesting the effectivity of these metrics to pick better quality drugs. Intriguingly, some of the Bayer-proprietary ligands (namely the indazoles 15, 16 and 17, as well as the “Scaffold II” compounds) were brought to our attention thanks to their respective kinetic- and enthalpic efficiency profiles. Further investigation of these compounds (beyond the scope of the present work) may inform about the potential utility of these novel optimization metrics in future drug discovery programs.

### Time-dependency of EZH2 inhibition in cancer cells

**Figure 5:**
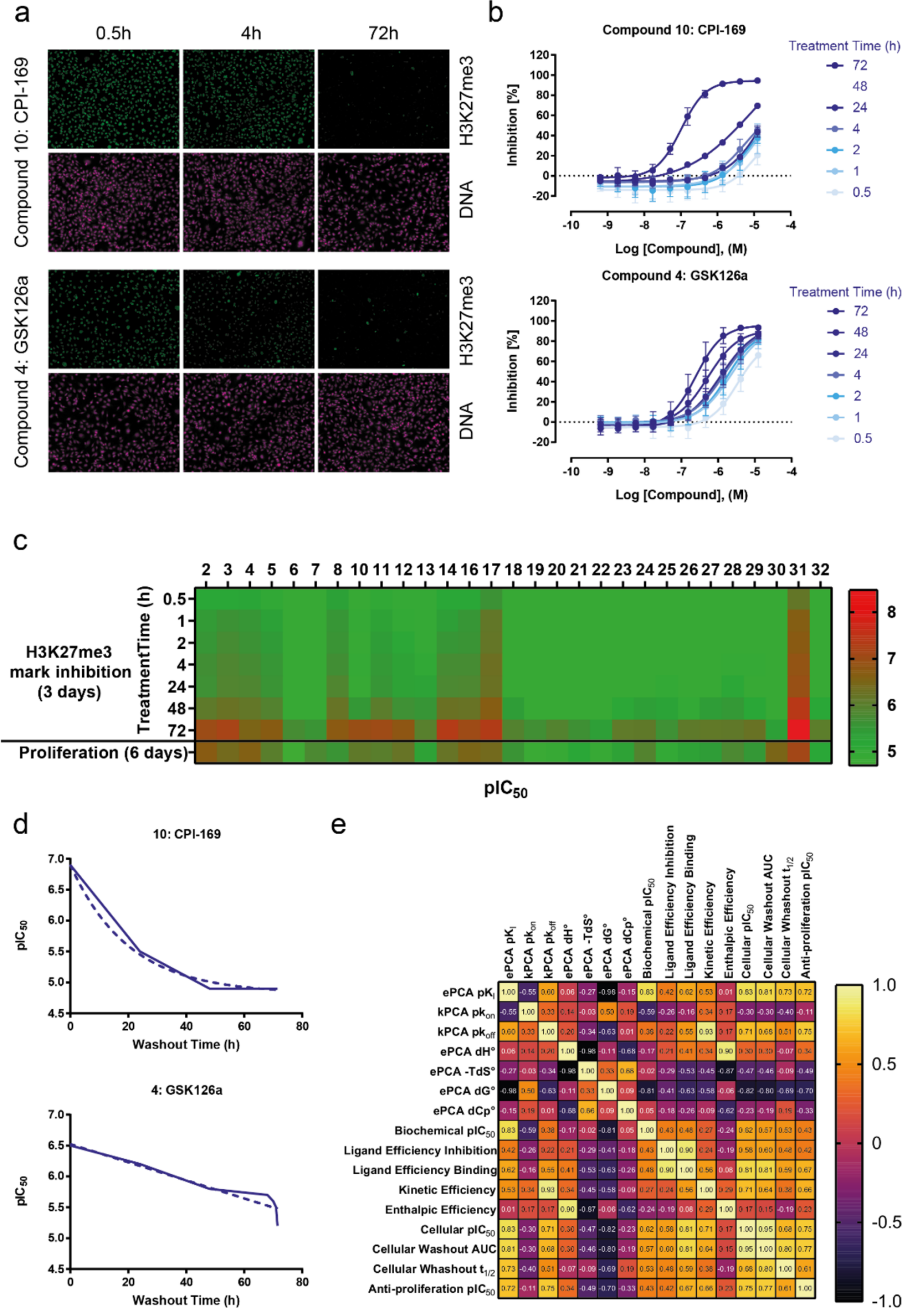
Time-dependent inhibition of Histone H3 Lys27 trimethylation in cells treated with pyridone-based active site EZH2 inhibitors. (**a**) Representative high-throughput fluorescence microscopy images of MDA-MB-231 cells treated with GSK126 and CPI-169, two representative EZH2 inhibitors from the indole class. Compounds were washed out upon different incubation times (pictures show three out of seven time points) and cells were fixed and stained with Hoechst 33342 for total DNA and a specific antibody recognizing the H3K27me3 mark. (**b**) Representative curves showing the inhibition H3K27me3 mark by increasing doses- and incubation times of the two compounds shown in (A). Normalized inhibition values were plotted versus the compound dose and the resulting curves were fitted to a 4-parameter logistic equation to derive the IC_50_ per incubation time values listed in the Supplementary Spreadsheet. (**c**) Overview of the time dependency of H3K27me3 inhibition by all EZH2 inhibitors investigated in this study. The heat map shows pIC_50_ values obtained at the different incubation times tested in the experiment (first seven rows). These values are compared with the KARPAS anti-proliferative effects of these compounds in a 6-day assay (bottom row). (**d**) Representative progression curves for GSK126 and CPI-169 reflecting the cellular potency of these compounds (y-axis) as a factor of the time cells were treated with them (x-axis). The solid lines show the actual data that served to calculate the area under the curve (AUC) for the potency decay with shorter incubation times. The dotted lines correspond to the fit to an exponential decay model, from which the washout half-life values (t_1/2_) were derived. (**e**) Spearman’s correlation matrix for the parameters investigated in this study. If data was acquired +/− H3K27me3 peptide, only the data with peptide is shown. Likewise, only biochemical inhibition with 15’ pre-incubation is represented. The color code in the heat map represents the Spearman’s coefficients, also given for the individual comparisons in the matrix.

Having confirmed the relationship between the interaction dynamics and the functional effect of EZH2 inhibitors in a cell-free assay, we next sought to evaluate whether these target binding properties would equally influence their pharmacological profiles in a disease-relevant cellular model. To this end, we configured the HCA assay previously reported by our group^38^ to perform washout experiments in which the exposure time of the compounds to the cells varied between half an hour and 3 days. As illustrated in Fig. 5a,b for the reference compounds 4 (GSK126a) and 10 (CPI-169), the histone trimethylation mark (H3K27me3) was robustly detected by confocal fluorescence microscopy and quantified in dose dependent fashion. These data allowed creating time dependent H3K27me3 mark inhibition profiles for nearly all compounds investigated in this study (Fig. 5c and Supplementary Spreadsheet).

To obtain further insights from these experiments, we plotted the cellular potencies measured with increasing wash out times and fitted the resulting curves to a single exponential decay model to determine a “washout half-life (t_1/2_)” for all inhibitors profiled. In addition (as previously shown by Georgi et al.^40^ for kinase inhibitors), we determined the area under the curve (AUC) for these treatment time-dependent pharmacological responses as a measure of the compound potency in a non-equilibrium system. Figure 5d shows examples of these time-dependent activity curves for the same reference compounds shown in Fig. 5a,b, as well as the corresponding fits to the models described above . The cellular washout t_1/2_ and AUC values computed for all compounds investigated are shown in the Supplementary Spreadsheet and served for comparison with other binding and activity parameters generated in this work (Figs. 5e and S8).

One burning question to these new data was whether there was a correlation between target residence time and cellular wash out t_1/2_ and AUC. Our comparison showed a significant correlation for most compounds (Fig. 5e), with CPI-169 and Bayer compound 8 being notable exceptions (Fig. S8a–b). CP-169 showed a relatively long target residence time but a moderate half-life in the cellular washout assay, while compound 8 exhibited relatively high t_1/2_ and AUC in cellular washout despite relatively fast k_off_ values. These observations led us to compare CPI-169 and compound 8 with similar compounds in a CaCo2 in vitro permeability assay (Supplementary Spreadsheet). The analysis revealed CPI-169 as the highest efflux ratio of all compounds tested, while GSK126a—the other indole in the study cohort—rather showed the opposite behavior. These results are a possible explanation for the CPI-169’s behavior, considering that the cellular model was an MDA-MB-231 breast cancer derived cell line which expresses some of the efflux transporters present in CaCo2. Intriguingly though, compound 8 displayed the fastest association rates in the cohort, suggesting that rebinding could play a role in its cellular mode-of-action, but it also exhibited the highest efflux ratio among the indazoles tested. In both cases, protracted biochemical (for CPI-169) or cellular (for compound 8) residence time do not translate into cellular efficacy (see below). These observations and apparent contradictions may merit further investigation, underscoring the importance of considering binding kinetics, cellular pharmacology and (micro) pharmacokinetics wholistically, to identify interesting compounds (from a PK/PD perspective) early in the drug discovery process.

As the goal of lead optimization campaigns is the identification of compounds with sufficient activity in cellular models for in vivo testing, we finally asked which among the several binding parameters characterized in this study would be a suitable predictor of in vitro cellular efficacy in a relevant model. To address this question, we measured the antiproliferative potencies of all compounds included in the study against the KARPAS-422 cell line (derived from B-cell non-Hodgkin lymphoma, one of the cancer types for which EZH2 inhibitors are being tested in clinical trials^2^) and compared all binding and activity data generated in the study with the resulting proliferation IC_50_ values both in a correlation matrix (Fig. 5e) and individually (exemplified for two parameters in Fig. S8c–d). In this analysis the best predictors for antiproliferative activity were the AUC values obtained from the cellular washout mechanistic assay measuring the H3K27me3 mark (*p* = 0.77), equally followed by the endpoint IC_50_’s from the same assay and—interestingly—by the k_off_ values extracted from ePCA and kPCA (both with *p* = 0.75). These observations are in line with recent report of EZH2 inhibitors being optimized according to their residence times^32,33^, providing further support for the important role binding kinetics studies can have in drug discovery^37^.

## Discussion

EZH2 is a clinically validated drug target in oncology and its inhibitors have been so far approved for follicular lymphoma and epithelioid sarcoma (Tazemetostat) or more recently also for adult T-cell leukemia/lymphoma (DS-3201/ Valemetostat)^62^. A positive therapeutic effect of EZH2 inhibition has also been postulated in other types of cancer, and clinical trials are ongoing for various EZH2 inhibitors in breast cancer (NCT05633979), metastatic prostate cancer (NCT04388852, NCT04846478, NCT03460977), small cell lung cancer (NCT03460977) and urothelial and kidney cancer (NCT04388852). Further optimizing EZH2 inhibitors towards optimal pharmacodynamic properties may allow to even extend the clinical benefit achievable with EZH2 targeting drugs. In this study we have developed and validated a suite of high throughput screening assays that enable large-scale mode of action profiling of EZH2 ligands during hit-to-lead and lead optimization campaigns with very low consumption of protein and other critical reagents.

Using these methods, we were able to reproduce various features of EZH2 biochemistry and molecular pharmacology, such as the allosteric activation by the H3K27me3 peptide and the competitive nature of the inhibition by pyridone-based inhibitors. We also confirmed based on a broader range of chemotypes that a protracted residence time augments compound potency in biochemical and cell-based test systems. Furthermore, we generated the largest set of structure kinetics relationships (SKR) data for EZH2 inhibitors reported to date. By conducting these assays with a disease relevant EZH2 mutant, we demonstrated the ability of the methodology to be expanded to further variants of the PRC2 complex.

One novel feature explored in this work was the thermodynamic characterization of EZH2 inhibitors, a nearly impossible task to be addressed with ITC, given the large quantities of protein required. Here we demonstrate that first generation of pyridone-based inhibitors have been optimized towards entropy-driven binding, an observation which is consistent with other drug classes investigated in the past^63^. It will be interesting to see whether a best-in-class generation of EZH2 drugs will rather be optimized for enthalpy-driven binding as it would be predicted from these previous analyses.

The non-equilibrium thermodynamics data presented in this paper represent a useful resource for molecular modelling efforts aiming at the design of better transition state analogues. These experiments allowed us to identify heat capacity changes in the complex formation phase as the most important constraint to binding affinity, strongly suggesting that highly effective EZH2 inhibitors fit more adequately to a pre-existing conformation of the enzyme rather than inducing a conformational change upon binding. In future work it will be interesting to compare this data set with thermodynamic data obtained in the presence of the H3K27me3 allosteric activator. It is tempting to speculate, that the role of this peptide is to push the equilibrium towards the conformation with the highest affinity for the SAM cofactor, which will consequently be more easily recognized by orthosteric inhibitors like the ones characterized in this study.

Another interesting aspect of the oncogenic role of EZH2 is the activity outside the PRC2 complex and the H3K27 methylation activity. Several non-canonical functions of EZH2 in the regulation of downstream gene expression have been described^64^. These can be divided into enzymatic (e.g. protein methylation of PLZF, RORα, GATA4, AR and STAT3) and non-enzymatic (scaffolding) functions of EZH2. It can be speculated that the guiding principles for the optimization of orthosteric inhibitors described in this study can be extended to other methylation substrates, as SAM remains the essential cofactor. It will be interesting to see in future studies how prolonged residence time affects new approaches to degrade EZH2 that may also target its non-enzymatic functions^65–67^.The binding parameters for wild type and Y462N mutant EZH2 resulting from our ePCA and kPCA correlated with biochemical inhibition. Furthermore, the slight impact of compound and enzyme pre-incubation on biochemical potency, suggest the usefulness of this qualitative approach to screen for slow binding EZH2 inhibitors. Of note, the sensitivity of this method could be even enhanced by adding saturating concentrations of the H3K27me3 allosteric modulator during the pre-incubation phase.

The cellular target engagement assay in washout format introduced in this study constitutes a valuable tool to understanding EZH2 pharmacology, especially if combined with in vitro and in vivo ADME/PK data. The results from these assays could be used in systems pharmacology modelling and prediction of the efficacious doses of EZH2 inhibitors in the non-equilibrium *in-vivo* systems. Finally, comparison of our anti-proliferation results to the target residence time data provides compelling evidence of the impact of the binding kinetic properties on the efficacy of EZH2 inhibitors in in vitro disease models. It is tempting to extend this conclusion to the in vivo situation, given the fact that the two compounds which are currently approved for therapeutic use exhibit the most protracted target binding. Based on recent reports of the impact of residence time characterization in EZH2 candidate generation programs^32,33^, we anticipate that the methods and data reported in this paper will become a source for study and inspiration in future drug discovery work on EZH2 and other targets.

## Materials and methods

### Synthesis of fluorescent EZH2 probe

#### General

All NMR spectra were recorded at ambient temperature on a 300 MHz Bruker DRX Spectrometer equipped with a 5 mm BBFO probe and a SampleExpress for automated sample handling. Proton (δH) chemical shifts are quoted in ppm and are internally referenced to the residual protonated solvent signal. Resonances are described as s (singlet), d (doublet), t (triplet) and so on. Coupling constants (J) are given in Hz and are rounded to the nearest 0.1 Hz.

Compound purity and identity were determined by LC–MS (Alliance 2795, Waters, Milford, MA). Purity was measured by UV absorbance at 210 nm. Mobile phase A consisted of 0.01% formic acid in water, while mobile phase B consisted of 0.01% formic acid in acetonitrile. The gradient ran from 5 to 95% mobile phase B over 1.75 min at 1.75 ml/min. An Agilent Poroshell 120 EC-C18, 2.7 µm, 3.0 × 30 mm column was used with column temperature maintained at 40 °C. 2.1 µL of sample solution were injected. RT refers to the retention time for the compound under the above conditions. Identity was determined on a SQ mass spectrometer by positive and negative electrospray ionization. m/z values are reported in Daltons, with the relevant fragment ions quoted in parentheses.

#### Experimental

##### 6-bromo-1-isopropyl-N-((6-methyl-2-oxo-4-propyl-1,2-dihydropyridin-3-yl)methyl)-1H-indazole-4-carboxamide


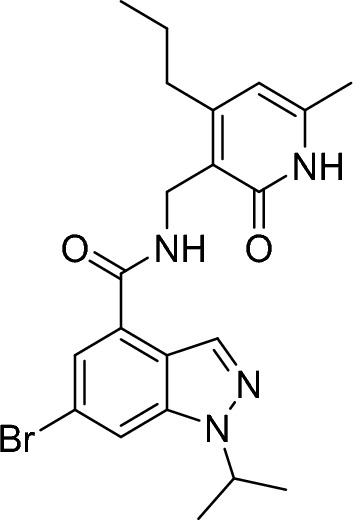


3-(aminomethyl)-6-methyl-4-propylpyridin-2(1H)-one hydrochloride (126 mg, 583 µmol, 1.00 eq.), N-ethyl-N-isopropylpropan-2-amine (418 µl, 2.33 mmol, 4.00 eq.) and PyBOP (364 mg, 699 µmol, 1.20 eq.) were added to a stirred solution of 6-bromo-1-isopropyl-1H-indazole-4-carboxylic acid (165 mg, 583 µmol, 1.00 eq.) in DMF (2.91 mL, 0.20 M). The resulting mixture was stirred at room temperature overnight and then dry loaded onto Celite. The residue was purified by flash column chromatography (0–20% methanol/dichloromethane gradient) to give the title compound (200 mg, 449 µmol, 77%).

RT = 1.49.

LC–MS m/z (ESI +) = 445 [M + H]^+^, 447 [M + H]^+^

^1^H NMR (300 MHz, Methanol-*d*_4_) δ 8.34 (s, 1H), 8.03 (s, 1H), 7.64 (s, 1H), 6.15 (s, 1H), 5.03–4.86 (m, 1H), 4.58–4.53 (m, 2H), 3.83–3.59 (m, 1H), 3.30–3.16 (m, 1H), 2.79–2.61 (m, 2H), 2.26 (s, 3H), 1.79–1.58 (m, 2H), 1.54 (d, *J* = 6.60 Hz, 3H), 1.36 (d, *J* = 6.60 Hz, 3H), 1.01 (t, *J* = 7.4 Hz, 3H).

##### Tert-butyl4-(5-(1-isopropyl-4-(((6-methyl-2-oxo-4-propyl-1,2-dihydropyridin-3-yl)methyl)carbamoyl)-1H-indazol-6-yl)pyridin-2-yl)piperazine-1-carboxylate


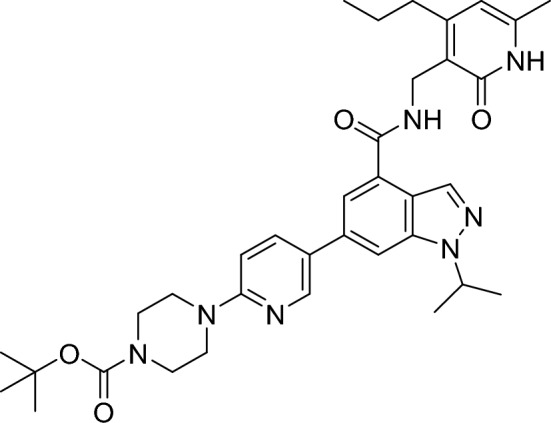


Xphos (25.0 mg, 52.0 µmol, 20 mol%), (6-(4-(tert-butoxycarbonyl)piperazin-1-yl)pyridin-3-yl)boronic acid (119 mg, 387 µmol, 1.50 eq.) and Xphos Pd G3 (44.0 mg, 52.0 µmol, 20 mol%) were added to a stirred suspension of 6-bromo-1-isopropyl-N-((6-methyl-2-oxo-4-propyl-1,2-dihydropyridin-3-yl)methyl)-1H-indazole-4-carboxamide (115 mg, 258 µmol, 1.00 eq.) in argon degassed THF (1.94 mL) and 0.50 M aqueous solution (0.65 ml). The resulting mixture was stirred at room temperature for 3 h and then dry loaded onto Celite. The residue was purified by flash column chromatography (0–20% methanol/dichloromethane gradient) to give the title compound.

RT = 1.62.

LC–MS m/z (ESI +) = 628 [M + H]^+^

##### 1-isopropyl-N-((6-methyl-2-oxo-4-propyl-1,2-dihydropyridin-3-yl)methyl)-6-(6-(piperazin-1-yl)pyridin-3-yl)-1H-indazole-4-carboxamide 2,2,2-trifluoroacetate


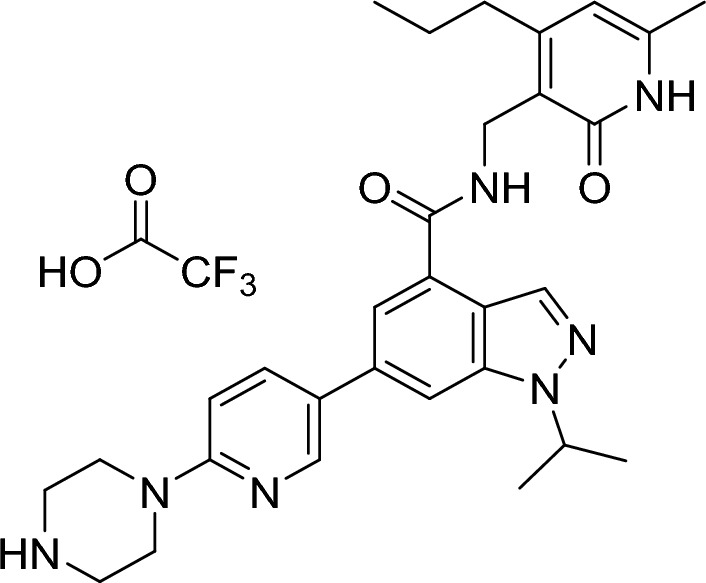


Trifluoroacetic acid (0.50 µl, 6.49 µmol, 0.08 eq.) and triethylsilane (25.4 µl, 159 µmol, 2.00 eq.) were added to a stirred solution of tert-butyl 4-(5-(1-isopropyl-4-(((6-methyl-2-oxo-4-propyl-1,2-dihydropyridin-3-yl)methyl)carbamoyl)-1H-indazol-6-yl)pyridin-2-yl)piperazine-1-carboxylate (50.0 mg, 80.0 µmol, 1.00 eq.) in dichloromethane (1.50 ml, 0.05 M). The resulting mixture was stirred at room temperature for 2 h and then concentrated under reduced pressure azeotroping 3 times with toluene. The crude material was used directly in the next step.

RT = 1.14.

LC–MS m/z (ESI +) = 528 [M + H]^+^

##### TERT-butyl (6-hydroxyhexyl) carbamate


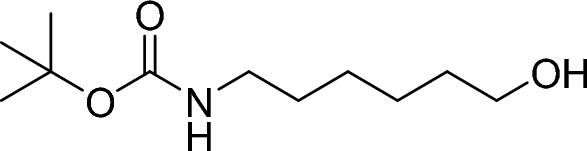


Di-tert-butyl dicarbonate (1.98 ml, 8.53 mmol, 1.00 eq.) was added to a solution of 6-aminohexan-1-ol (1.00 g, 8.53 mmol, 1.00 eq.) in ethanol (17.1 ml, 0.50 M) stirred at 0 °C. The resulting mixture was warmed to room temperature and stirred overnight. Additional 200 mg di-tert-butyl dicarbonate were added to the mixture and stirred for 10 h. The mixture was concentrated under reduced pressure and azetroped 3 times from toluene. The crude material was used directly in the next step.

RT = 1.12.

LC–MS m/z (ESI +) = 162 [M + H]^+^

##### Tert-butyl (6-oxohexyl) carbamate


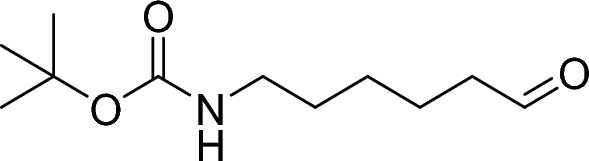


Pyridinium chlorochromate (480 mg, 2.23 mmol, 1.51 eq.) was added to a stirred solution of tert-butyl (6-hydroxyhexyl) carbamate (320 mg, 1.47 mmol, 1.00 eq.) I n dichloromethane (12.0 ml, 0.12 M), followed by addition of neutral alumina (1.20 g, 11.8 mmol, 8.00 eq.). The resulting mixture was stirred at room temperature for 5 h and then diluted with dichloromethane and filtered. The filtrate was washed twice with water, dried (sodium sulfate), filtered and concentrated under reduced pressure. The residue was purified by flash column chromatography (15–30% ethyl acetate/hexanes gradient) to give the title compound (110 mg, 511 µmol, 35%).

^1^H NMR (300 MHz, Chloroform-*d*) δ 9.76 (t, *J* = 1.7 Hz, 1H), 3.11 (q, *J* = 6.6 Hz, 2H), 2.44 (td, *J* = 7.3, 1.7 Hz, 2H), 1.65 (dt, *J* = 14.6, 7.2 Hz, 2H), 1.56–1.19 (m, 16H).

##### Tert-butyl(6-(4-(5-(1-isopropyl-4-(((6-methyl-2-oxo-4-propyl-1,2-dihydropyridin-3-yl)methyl)carbamoyl)-1H-indazol-6-yl)pyridin-2-yl)piperazin-1-yl)hexyl)carbamate


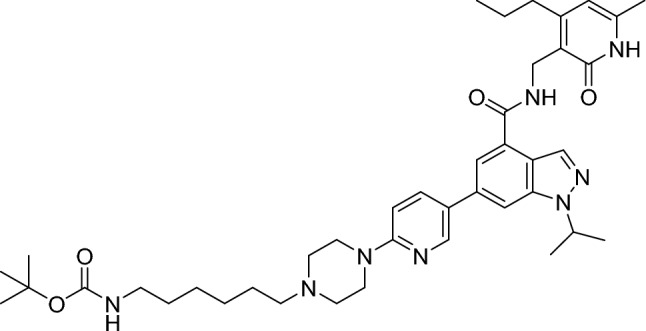


Tert-butyl (6-oxohexyl)carbamate (34.4 mg, 160 µmol, 2.00 eq.) was added to a stirred solution of crude 1-isopropyl-N-((6-methyl-2-oxo-4-propyl-1,2-dihydropyridin-3-yl)methyl)-6-(6-(piperazin-1-yl)pyridin-3-yl)-1H-indazole-4-carboxamide 2,2,2-trifluoroacetate (51.3 mg, 80.0 µmol, 1.00 eq.) in dichloromethane (800 µl, 0.10 M), followed by addition of sodium triacetoxyborohydride (45.8 mg, 216 µmol, 2.70 eq.). The resulting mixture was stirred at room temperature for 90 min. The mixture was diluted with sat. aq. sodium bicarbonate solution and then extracted 3 times with ethyl acetate. The combined organic extracts were washed with brine, dried (sodium sulfate), filtered and concentrated under reduced pressure. The residue was purified by flash column chromatography (15–35% ethyl acetate/hexanes gradient) to give the title compound (22.0 mg, 30.0 µmol, 38%).

RT = 1.38.

LC–MS m/z (ESI +) = 727 [M + H]^+^

^1^H NMR (300 MHz, Chloroform-*d*) δ 12.70 (s, 1H), 8.56–8.32 (m, 2H), 8.00 (t, *J* = 5.8 Hz, 1H), 7.82–7.67 (m, 2H), 7.58 (s, 1H), 6.67 (d, *J* = 8.9 Hz, 1H), 5.92 (s, 1H), 4.96–4.79 (m, 1H), 4.66 (d, *J* = 5.7 Hz, 2H), 4.56 (s, 1H), 3.67–3.53 (m, 4H), 3.47 (q, *J* = 7.0 Hz, 1H), 3.11 (q, *J* = 6.9 Hz, 2H), 2.70 (t, *J* = 7.7 Hz, 2H), 2.61–2.49 (m, 4H), 2.38 (t, *J* = 7.7 Hz, 2H), 2.15 (s, 3H), 1.68–1.15 (m, 23H), 1.00 (t, *J* = 7.3 Hz, 3H), 0.87 (t, *J* = 6.5 Hz, 1H).

##### 6-(6-(4-(6-aminohexyl)piperazin-1-yl)pyridin-3-yl)-1-isopropyl-N-((6-methyl-2-oxo-4-propyl-1,2-dihydropyridin-3-yl)methyl)-1H-indazole-4-carboxamide


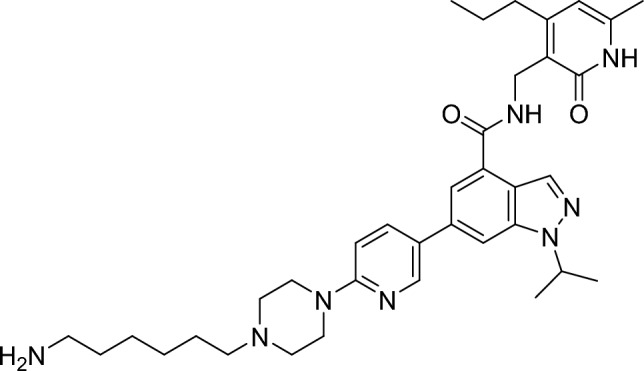


Trifluoroacetic acid (0.94 mg, 8.25 µmol, 0.30 eq.) and triethylsilane (6.40 mg, 55.0 µmol, 2.00 eq.) were added to a stirred solution of tert-butyl (6-(4-(5-(1-isopropyl-4-(((6-methyl-2-oxo-4-propyl-1,2-dihydropyridin-3-yl)methyl)carbamoyl)-1H-indazol-6-yl)pyridin-2-yl)piperazin-1-yl)hexyl)carbamate (20.0 mg, 28.0 µmol, 1.00 eq.) in dichloromethane (0.90 ml, 0.30 M). The resulting mixture was stirred at room temperature for 2 h and then concentrated under reduced pressure azeotroping with toluene. The residue was suspended in sat. aq. sodium bicarbonate and extracted with dichloromethane. The organic layer was dried (magnesium sulfate), filtered and concentrated under reduced pressure. The residue was purified by flash column chromatography (0–20% MeOH/DCM with 1% NH_3_ gradient) to give the title compound (16.0 mg, 26.0 µmol, 93%).

RT = 1.01.

LC–MS m/z (ESI +) = 627 [M + H]^+^

^1^H NMR (300 MHz, Methanol-*d*_4_) δ 8.58 (d, *J* = 2.5 Hz, 1H), 8.36 (s, 1H), 8.04 (dd, *J* = 8.9, 2.6 Hz, 1H), 7.93 (s, 1H), 7.76 (s, 1H), 7.02 (d, *J* = 8.8 Hz, 1H), 6.14 (s, 1H), 5.17–5.01 (m, 1H), 4.60 (s, 2H), 3.91–3.45 (br s, 3H), 3.27–3.14 (m, 4H), 2.94 (t, *J* = 7.6 Hz, 2H), 2.73 (t, *J* = 7.6 Hz, 2H), 2.26 (s, 3H), 1.90–1.75 (m, 2H), 1.62 (dd, *J* = 28.4, 7.0 Hz, 17H), 1.52–1.40 (m, 4H), 1.01 (t, *J* = 7.3 Hz, 3H).

##### 2-[(1E,3E)-5-[(2Z)-3,3-dimethyl-1-{5-[(6-{4-[5-(4-{[(6-methyl-2-oxo-4-propyl-1,2-dihydropyridin-3-yl)methyl]carbamoyl}-1-(propan-2-yl)-1H-indazol-6-yl)pyridin-2-yl]piperazin-1-yl}hexyl)carbamoyl]pentyl}-5-sulfo-2,3-dihydro-1H-indol-2-ylidene]penta-1,3-dien-1-yl]-1-(2-methoxyethyl)-3,3-dimethyl-5-sulfo-3H-indol-1-ium trifluoroacetic acid trifluoroacetate


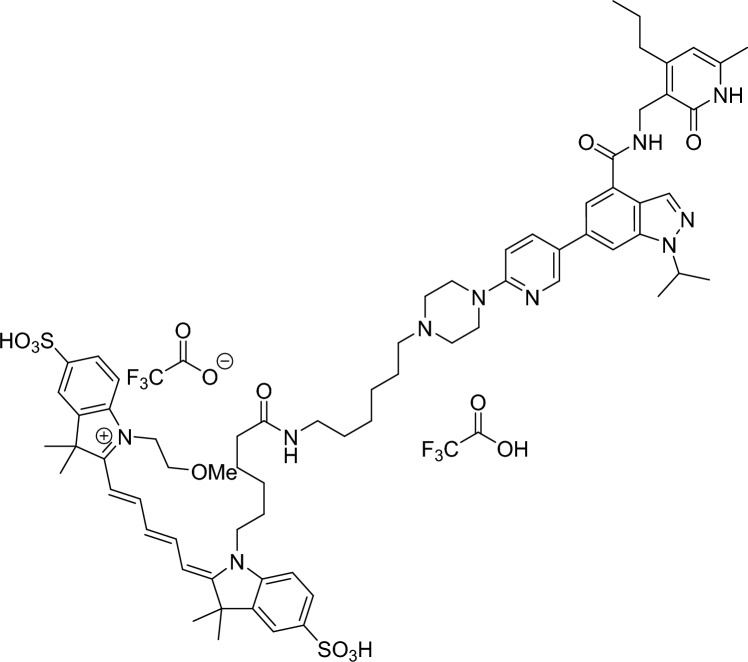


DIEA (5.42 µl, 31.0 µmol, 5.00 eq.) was added to a stirred solution of 6-(6-(4-(6-aminohexyl)piperazin-1-yl)pyridin-3-yl)-1-isopropyl-N-((6-methyl-2-oxo-4-propyl-1,2-dihydropyridin-3-yl)methyl)-1H-indazole-4-carboxamide (3.89 mg, 6.20 µmol, 1.00 eq.) in DMF (388 µl, 0.016 M), followed by addition of sodium 2-((1E,3E,5Z)-5-(1-(6-((2,5-dioxopyrrolidin-1-yl)oxy)-6-oxohexyl)-3,3-dimethyl-5-sulfonatoindolin-2-ylidene)penta-1,3-dien-1-yl)-1-(2-methoxyethyl)-3,3-dimethyl-3H-indol-1-ium-5-sulfonate (5.00 mg, 6.20 µmol, 1.00 eq.). The resulting mixture was then stirred at room temperature for 30 min. The reaction mixture was directly purified by reverse phase column chromatography (0–100% acetonitrile/water with 0.01% TFA gradient) to give the title compound (3.30 mg, 2.54 µmol, 41%).

RT = 0.96.

LCMS m/z (ESI +) = 1296 [M + H]^+^

Instrument: Waters Acquity UPLC-MS SQD 3001; Column: Acquity UPLC BEH C18 1.7 µ 50 mm × 2.1 mm; Eluent A: Water + 0.1% Formic Acid, Eluent B: Acetonitrile; Gradient: 0–1.6 min 1–99% B, 1.6–2.0 min 99% B; Flow rate: 0.8 ml/min; Temperature: 60 °C; Injection: 2 µl; DAD scan: 210–400 nm; ELSD.

#### Cloning, expression, and purification of 5-component recombinant PRC2

Recombinant PRC2 complex with EZH2^+/+^ was purchased from Active Motif. Synthetic cDNA encoding N-terminal 6His-tagged full length EZH2^Y641N^, EZH2^Y111L^, EZH2^Y111D^, EZH2^Y641N, Y661DEZH2^, EZH1, N-terminal 6His-tagged SUZ12, N-terminal 6His-tagged RBBP4, N-terminal 6His-tagged AEBP2 and N-terminal FLAG-tagged EED were codon-optimized by Eurofins for expression in insect cells, subcloned into pDONR221 and subsequently recombined with a modified pDEST by Gateway cloning (Invitrogen)*.* The flashback Gold (Oxford Expression Technologies,) expression system was used to generate baculoviruses for co-infection in Sf9 cells. The harvested cells were resuspended in lysis buffer containing 20 mM Tris–HCl (pH 8.0), 500 mM NaCl, 20% glycerol, 4 mM MgCl_2_, 0.4 mM ETDA, 2 mM DTT, 0.05% NP40, benzonase and incubated on ice for 30 min. The lysate was clarified by centrifugation and incubated in batch with anti-FLAG M2 beads (Sigma-Aldrich) for 4 h at 4 °C. Beads were filled in a Kronlab glass column (YMC Europe) and washed with lysis buffer and buffer B containing 20 mM HEPES (pH 7.9), 2 M KCl, 0.4 mM EDTA, 10% glycerol, 0.5 mM DTT, 0.05% NP40. The bound protein complex was eluted with elution buffer containing 20 mM HEPES (pH 7.9), 300 mM KCl, 0.4 mM EDTA, 10% glycerol, 0.5 mM DTT, 0.05% NP40 and 150 µg/ml FLAG peptide. For chemical biotinylation recombinant PRC2 was buffer exchanged in 20 mM HEPES (pH 7.9), 100 mM KCl, 10% glycerol, 0.5 mM DTT using a HiPrep 26/10 desalting column (GE Healthcare).

#### Biotinylation of PRC2

PRC2 was chemically biotinylated by using EZ-Link sulfo-NHS-LC-Biotin (ThermoFisher) and following the instructions provided by the kit manufacturer. The recombinant PRC2 was buffer exchanged to 20 mM HEPES (pH 7.9), 100 mM NaCl, 0.5 mM DTT by dialysis and the protein-biotin ratio was determined using a HABA dye-based quantification kit (ThermoFisher). Biotinylation was verified by Western blot using avidin conjugated to alkaline phosphatase (Sigma-Aldrich) and by tryptic in-gel digestion of PRC2 proteins and nanoLC-MS/MS analysis. Tryptic peptides were searched against an in-house protein database considering a carbamidomethylation of cysteines as fixed modification and methionine oxidation and sulfo-NHS-LC-biotin modified lysines as variable modification using Mascot (Matrix Science).

#### TR-FRET assays

Biotinylated PRC2 complexes and the fluorescent probe used in TR-FRET assays were generated as described in the supplementary methods section. All assays were performed in aqueous assay buffer composed of 0.01 M HEPES [pH 7.4], 100 mM NaCl, 2.75 mM Dithiothreitol (DTT), 0.01% bovine serum albumin (BSA), and 0.01% pluronic F-127 on black “small volume” 384-well microtiter plates (Greiner). TR-FRET signals were recorded in a PHERAstar FS microtiter plate reader using the standard settings for HTRF (excitation: 340 nm, donor emission: 622 nm, and acceptor emission: 665 nm).

Optimum enzyme concentrations and equilibrium affinity of the fluorescent probe were determined by cross-titrating 4-step twofold dilutions of the (1:1 molar ratio) SA-Tb-labeled enzyme (0.25–4 nM) and the probe (12-step, 0–20 nM for EZH^WT^ or 0–500 nM for EZH2^Y641N^) in a final volume of 10 µL. Plates were spun down (30 s, 800 g) and incubated for 2 h at RT and overnight at 4 °C prior to signal acquisition. Subsequent experiments were performed at 0.5 nM EZH2^+/+^ and 1 nM EZH2^Y641N^ labeled with the SA-Tb TR-FRET donor (Cisbio) in 1:1 molar ratio.

The allosteric binding affinity (K_b_) of the synthetic H3K27me3-derived activator peptide (ATKAAR-[K-Me3]-SAPATGGVKKPHRYRPGGK-amide, Biosyntan) was determined by cross-titrating the peptide at 8 concentrations ranging from 20 nM to 25 µM in fourfold dilution steps, and the fluorescent probe as described above. TR-FRET signals were recorded at room temperature upon short centrifugation and incubation intervals of 1 h, 2 h, 4 h, 6 h and overnight (at 4 °C).

To measure association kinetics of the probe, the first syringe of the PHERAstar FS injection device was used to rapidly dispense 3 µl of SA-Tb-labeled protein (0.5 nM for EZH2^+/+^, 1 nM for EZH2^Y641N^) to 2 µl of fluorescent probe at increasing concentrations (0–10 nM for EZH2^+/+^ or 0–120 nM for EZH2^Y641N^, 8-step, twofold dilution). After recording association (100 cycles in 10 s intervals), probe dissociation rates were analyzed by displacement (“chase”) of the probe with unlabeled probe. To this end, 5 µl of the unlabeled ligand were added with the second syringe to concentrations of 1 µM for PRC2/EZH2+/+ and 3 µM for PRC2/EZH2^Y641N^ in a final volume of 10 µl. TR-FRET signals were acquired in 160 kinetic cycles with 15 s (EZH2^+/+^) or 60 s (EZH2^Y641N^) intervals. The allosteric effector role of H3K27me3 was evaluated by adding the peptide at final concentrations of 1.5 µM and 10 µM for EZH2^Y641N^ and EZH2^+/+^, respectively. To this end, both peptide and enzyme complexes were pre-incubated at RT for 2 h prior to the start of the kinetic experiment. For non-specific background signals, the same number of samples was processed in the presence of an excess of unlabeled probe (1 µM for PRC2/EZH2^+/+^ and 3 µM for PRC2/EZH2^Y641N^).

Evaluation of unlabeled compounds in equilibrium conditions was conducted in assay-ready plates containing 50 nl of compound dilutions (0–20 µM, 12-step 3.5-fold dilution) or DMSO as vehicle control. Plates were filled with 2 µl of the SA-Tb-labeled protein (0.5 and 1 nM for EZH2^Y641N^ and EZH2^+/+^, respectively, or assay buffer in inhibition control wells) and 3 µl of fluorescent probe (5 and 30 nM for EZH2^Y641N^ and EZH2^+/+^, respectively) using a Multidrop Combi liquid dispenser (Thermo Fischer Scientific). After short centrifugation and incubation intervals of 1 h, 2 h, 3 h (at RT) and overnight (at 4 °C), plates were brought to room temperature and TR-FRET signals were recorded.

Kinetic evaluation of unlabeled compounds was performed in ready-to-use plates containing 100 nl of compound dilutions (0–2.5 µM, 5-step tenfold dilution) or DMSO as vehicle control. Plates were filled with 5 µl of fluorescent probe (5 and 30 nM for EZH2^Y641N^ and EZH2^+/+^, respectively, or assay buffer in inhibition control wells) and brought to the plate reader, where the first syringe of the injection system (previously rinsed with NaOH and H_2_O) had been primed 3 times with 500 µl of Tb-labeled protein, and the second syringe had been primed 3 times with 500 µl of dissociation solution (1 µM unlabeled probe for EZH2^+/+^, 3 µM unlabeled probe for EZH2^Y641N^). The experiments were started by adding 5 µl of the Tb-labeled protein with the first syringe and the TR-FRET signals were recorded over time during 600 s (EZH2^+/+^) or 800 s (EZH2^Y641N^) with kinetic intervals of 10 s.

For thermodynamic analysis of the binding to EZH2+/+ the probe characterization, equilibrium and kinetic probe competition assays described above (previously conducted at 25 °C) were performed at 5, 15, 30 and 37 °C. For the first two temperatures, the PHERAstar FS plate reader was installed in a chromatography refrigerator and pre-equilibrated to the desired temperature for at least 16 h before measurements were conducted. For measurements at 25, 30 and 37 °C, the temperature control functionality of the instrument was used.

#### Scintillation proximity assays

Scintillation proximity assay (SPA) was performed with PRC2 containing subunits EZH1, EZH2^+/+^, EZH2^Y641N^, EZH2^Y111L^, EZH2^Y111D^ or EZH2^Y641N,Y661D^ (see supplementary methods section for protein production protocols) at final concentrations of 1.25, 0.7, 0.9, 1.0, 1.0 and 3.0 ng/µl, in assay buffer (H_2_O, 0.01 M HEPES [pH 7.4], 2.75 mM DTT, supplemented with 0.01% BSA and 0.01% pluronic F-127). Assay-ready plates containing 50 nl of compound dilutions and DMSO controls were filled with 2.5 µL enzyme solution and pre-incubated for 15 min. Subsequently, a 2.5 µL substrate mix containing 15 µM of the biotinylated (Btn) H3K27 substrate peptides ARTKQTAR-K(me3)-STGGKAPRKQLATKAAR-KSAPATGGVKKPHR-K(Btn)-amide (for EZH1, EZH2^+/+^, EZH2^Y111L^ and EZH2^Y111D^) and ARTKQTARKSTGGKAPRKQLATKAAR-K(me2)-SAPATGGVKKPHR-K(Btn)-amide (for EZH2^Y641N^ and EZH2^Y641N^) (Biosyntan), 125 nM of ^3^H-SAM and 875 nM SAM (Sigma Aldrich) was added and incubated for 60 min (EZH1, EZH2^+/+^, EZH2^Y111L^, EZH2^Y111D^ and EZH2^Y641N,Y661D^) or for 90 min (EZH2^Y641N^). Experiments were also performed without pre-incubation of enzyme and compounds. In this case, the substrate solution was added before enzyme. The reaction was stopped using 1.6 µg/µl streptavidin beads (Perkin Elmer) and 700 µM of SAM (Perkin Elmer). The plates were sealed with transparent foil. The signals were measured after 1 h using ViewLux plate reader (Perkin Elmer) at wavelength of 620 nm.

#### High content analysis of cellular PRC2/EZH2 inhibition

MDA-MB-231 cells purchased from the DSMZ (75 cells/µl) were incubated at 37 °C in F12 mix medium for 24 h until adherence. Cells were treated with EZH2 inhibitor compounds in decreased threefold dilution series at 10 concentrations ranged from 12.4 µM to 0.631 nM (dilution contains 0.6% DMSO). In the low- and high-signal control wells, test compounds were replaced by 10 µM of EZH2 inhibitor (equal to unlabeled probe from ePCA and kPCA) or DMSO, respectively. At various time points (0.5, 1, 2, 4, 24, 48 or 72 h), medium containing the compounds was discarded, the cells were washed three times with medium and incubated with fresh medium (15 µl/well) at 37 °C until the endpoint of 72 h. After 72 h, the medium was removed; the cells were washed with phosphate buffer saline (PBS) and fixed using 4% paraformaldehyde (PFA) for 1 h at RT. After fixation, the cells were washed with PBS and incubated with 1% BSA blocking solution at RT for 1 h. Blocking solution was discarded, primary monoclonal antibody rabbit Tri-methyl-histone H3K27 (C36B11, Cell signaling) diluted 1:200 in blocking solution was added and incubated overnight at 4 °C. Antibody solution was rejected, the cells were washed with PBS, treated with secondary antibody Goat-Anti-Rabbit-Cy2 Alexa 488 (1.5 mg/ml, 111 546 047, Jackson Immuno Research) diluted 1:200 in blocking solution and incubated at RT for 1 h. Cells were further stained for 20 min by adding Hoechst 33,342 (1 mg/ml, Life Technologies) as counterstain for all nuclei at a final dilution of 1:2500. Afterwards, cells were washed three times with PBS and incubated at 4 °C until measuring. Fluorescence signals were captured by an Opera Phenix™ High Content Screening (HCA) system (Perkin Elmer) by 10× magnification. Images (5 sites per well) were acquired at two different wavelength channels (Hoechst at 405 nm and Cy2 at 488 nm)^34^. Microscopy images were initially visualized and preprocessed with MetaXpress High Content Image Acquisition & Analysis Software (Molecular Devices) as previously described^34^. Raw data import, normalization, quality control and fitting curves for IC_50_ determination of tested compounds were done with Genedata Screener^®^ for high-content screening. Wells with no recognizable signals were masked and the average intensity of the trimethylation signal was normalized to the DMSO control (0%). IC_50_ values derived from single incubation durations were normalized to IC_50_ at 72 h or 24 h and plotted against incubation time to calculate the cellular t_1/2_ and AUC values with the “one phase decay” and “AUC” models available in the Graph Pad Prism Analysis Software.

#### Cellular proliferation assay

Cell viability was determined by means of the AlamarBlue^®^ reagent (Thermo Fisher Scientific) following instructions from the manufacturer. Briefly, KARPAS-422 cells (Public Health England, Catalogue No.: 06101702) were seeded at a concentration of 2000 cells/well in 100 µl of growth medium (RPMI1640, 20% FCS) on 96-well microtiter plates. The plates were treated with various compound dilutions (1E-5 M, 3E-6 M, 1E-6 M, 3E-7 M, 1E-7 M, 3E-8 M, 1E-8 M, 3E-9 M) and incubated at 37 °C for 144 h prior to staining and fluorescence signal acquisition with a Victor X3 Multilabel Plate Reader (Perkin Elmer). Anti-proliferative IC_50_s were calculated by fitting the normalized AlamarBlue® fluorescence values to a 4-parameter logistic equation using the Electronic Notebook (ELN) software from IDBS.

#### Permeability assay

Caco2 cells purchased from the DSMZ were seeded at a density of 4.5 × 10^4^ cells per well and grown for 15 d in DMEM with typical supplements. Cells were kept at 37 °C in a humidified 5% CO_2_ atmosphere. Before the permeation assay was run, the culture medium was replaced by an FCS-free HEPES carbonate transport buffer (pH 7.2). For assessment of monolayer integrity, the transepithelial electrical resistance was measured. Test compounds were pre-dissolved in DMSO and added either to the apical or basolateral compartment at a final concentration of 2 µM. Before and after 2 h incubation at 37 °C, samples were taken from both compartments. Analysis of compound content was conducted after precipitation with methanol by LC/MS/MS analysis. Reference compounds were analyzed in parallel as assay control. Permeability (Papp) was calculated in the apical to basolateral (A → B) and basolateral to apical (B → A) directions. The efflux ratio basolateral (B) to apical (A) was calculated by dividing Papp B–A by Papp A–B.

### Data analysis

#### General statistics

Mean values and standard deviations (SD) of all parameters reported in this study were calculated from at least two independent experiments (N = 2), each performed in replicate microtiter plates containing samples in duplicate (n = 4). The confidence intervals of the binding parameters described were derived from the nonlinear models used to analyze the data. Spearman and Pearson models available in the Graph Pad Prism Analysis Software were used for correlation matrix analysis.

#### Fluorescent probe characterization assays

For fluorescent probe affinity (K_D_) determination, blank-subtracted TR-FRET signals from equilibrium binding saturation experiments were evaluated using the “one site-specific binding” model of the GraphPad Prism (version 6.07 for Windows) software. The affinity constant for the allosteric binding of the H3K27me3 peptide (K_b_) was analyzed using the model “Allosteric EC50 shift”^42^ Y = Bottom + (Top–Bottom)/(1 + (EC50*Antag/X)^HillSlope), where Antag = (1 + B/KB)/(1 + alpha*B/KB). To characterize the kinetic association (k_1_) and dissociation (k_2_) constants of the fluorescent probe, blank-subtracted TR-FRET traces were fitted to the GraphPad Prism model “association, then dissociation kinetics of two or more concentrations of hot”.

#### Equilibrium competition assays

For equilibrium binding competition (ePCA) and SPA enzyme inhibition assays, raw data import, quality control, normalization, and curve fitting to a 4-parameter logistic equation for IC_50_ determination were performed using the Genedata Screener^®^ software (Genedata AG). ePCA K_i_ values were either calculated using the Cheng-Prussof equation^68^ or with the GraphPad Prism model “One-site—Fit K_i_”.

#### Kinetic competition assays

TR-FRET kPCA traces were exported via Microsoft Excel to GraphPad Prism, where non-specific signals were subtracted and kinetic association competition traces were fitted to the Motulsky–Mahan model “kinetics of competitive binding” to obtain k_on_ (k_3_), k_off_ (k_4_) and K_D_ parameters.

#### Thermodynamics binding assays

Equilibrium and kinetic binding constants obtained at various temperatures were used to determine the thermodynamic parameters by van’t Hoff and Eyring analysis, as described elsewhere for SPR and radioligand binding experiments^46,69^. For the van’t Hoff thermodynamic analysis**,** changes in entropy ΔS° and enthalpy ΔH° were estimated by linear regression using the van’t Hoff equation.

$${\text{ln}} {\text{K}}_{{\text{A}}} = \Delta {\text{S}}^\circ {\text{/R}} - \Delta {\text{H}}^\circ /\left( {{\text{RT}}} \right),$$

where K_A_ = 1/K_D_ = k_on_/k_off_ is the equilibrium constant, R is the universal gas constant (8.314 J mol^−1^ K^−1^) and T the absolute temperature in K.

Changes in Gibb’s free energy ΔG° were calculated according to.

$$\Delta {\text{G}}^\circ \, \left( {\text{T}} \right)\, = \,\Delta {\text{H}}^\circ \, - \,{\text{T}}\, \times \,\Delta {\text{S}}^\circ$$

Additionally, the possible temperature dependence of the thermodynamic parameters was considered using the integrated form of the van’t Hoff equation^47^ for nonlinear regression to yield the changes in entropy, enthalpy, and heat capacity ΔC_p_:

$${\text{ln K}}_{{\text{A}}} \, = \, - \,\Delta {\text{H}}^\circ \left( {{\text{T}}^\circ } \right)/\left( {{\text{RT}}} \right)\, + \,\Delta {\text{S}}^\circ \left( {{\text{T}}^\circ } \right)/{\text{R}}\, + \,\Delta {\text{C}}_{{\text{p}}} /{\text{R}}[\left( {{\text{T}}\, - \,{\text{T}}^\circ } \right)/{\text{T}}\, - \,{\text{ln }}\left( {{\text{T}}/{\text{T}}^\circ } \right)],$$

where T° is the reference temperature 298.15 K, and ΔS°(T°) and ΔH°(T°) the entropy and enthalpy changes at this reference temperature.

For Eyring transition state analysis, changes in the activation entropy ΔS^#^ and enthalpy ΔH^#^ were estimated by Eyring analysis using linear regression of.

$${\text{ln}} \left( {{\text{kh}} / \left( {{\text{k}}_{{\text{B}}} {\text{T}}} \right)} \right)\, = \,\Delta {\text{S}}^{\# } /{\text{R}}\, - \,\Delta {\text{H}}^{\# } /({\text{RT}}).$$

where k is the kinetic rate constant, h is Plank’s constant (6.626 × 10^−34^ Js), k_B_ is Boltzmann’s constant (1.381 × 10^−23^ JK^−1^), T the absolute temperature in K and R is the universal gas constant.

To consider the possible temperature dependence, the activation entropy and enthalpy were obtained along with the activation heat capacity ΔC^#^_p_ by nonlinear regression using.

$${\text{ln}} \left( {{\text{kh}} / \left( {{\text{k}}_{{\text{B}}} {\text{T}}} \right)} \right) = - \,\Delta {\text{H}}^{\# } \left( {{\text{T}}^\circ } \right)/\left( {{\text{RT}}} \right)\, + \,\Delta {\text{S}}^{\# } \left( {{\text{T}}^\circ } \right)/{\text{R}}\, + \,\Delta {\text{C}}^{\# }_{{\text{p}}} /{\text{R}}[\left( {{\text{T}}\, - \,{\text{T}}^\circ } \right)/{\text{T}}\, - \,{\text{ln }}\left( {{\text{T}}/{\text{T}}^\circ } \right)],$$

where T° is the reference temperature 298.15 K, and ΔS^#^(T°) and ΔH^#^ (T°) the activation entropy and enthalpy changes at this reference temperature.

#### Ligand efficiency metrics

To compute ligand efficiencies pKi and pIC_50_ values from ePCA and activity assays were used to calculate ΔG (ΔG = 1.4*pIC_50_ or 1.4*pKi) and this value was divided by the number of non-hydrogen atoms as described by Schultz^51^. Kinetic-^60^ and enthalpic efficiencies^70^ were computed by dividing target residence times (RT) and binding enthalpies (ΔH) by their number of non-hydrogen atoms.

### Supplementary Information

Supplementary Information 1.

Supplementary Information 2.

## Data Availability

The datasets used and/or analyzed during the current study are available from the corresponding author on reasonable request.
