# Supplementary material for: A miniaturized mode-of-action profiling platform enables high throughput characterization of the molecular and cellular dynamics of EZH2 inhibition
Source: Sci Rep. 2024 Jan 19;14:1739. doi: 10.1038/s41598-023-50964-x (PMC10799085; doi:10.1038/s41598-023-50964-x)
Supplement: Supplementary file 1 — Supplementary Information 1. [file 41598_2023_50964_MOESM1_ESM.pdf]

Supplementary Information

A miniaturized mode-of-action profiling platform enables high throughput characterization of the molecular and cellular dynamics of EZH2 inhibition

Lilia Falkenstern[1,3,8], Victoria Georgi[1,4,8], Stefanie Bunse[1,4], Volker Badock[1,4], Manfred Husemann[1], Ulrike Roehn[1,4], Timo Stellfeld[1,4], Mark Fitzgerald[1,5], Steven Ferrara[2], Detlef Stöckigt[1,4], Carlo Stresemann[1,4], Ingo V. Hartung[1,6], and Amaury Fernández-Montalván[1,7,9]

[1] Bayer AG, Müllerstrasse 178, 13353 Berlin, Germany

[2] Broad Institute, Merkin Building, 415 Main St, Cambridge, MA 02142, USA

[3] Present address: Rentschler Biopharma SE, Erwin-Rentschler-Straße 21, 88471 Laupheim, Germany

[4] Present address: Nuvisan Innovation Campus Berlin, Müllerstrasse 178, 13353 Berlin, Germany

[5] Present address: Nested Therapeutics, 1030 Massachusetts Avenue, Suite 410 Cambridge, MA 02138, USA

[6] Present address: Merck KGaA Frankfurter Str. 250, 64293 Darmstadt, Germany

[7] Present address: Boehringer Ingelheim Pharma GmbH & Co. KG, Birkendorfer Str. 65, 88400 Biberach an der Riß, Germany

[8] Equal contributions

[9] Correspondence to amaury.fernandez@boehringer-ingelheim.com

Supplementary Figures

a

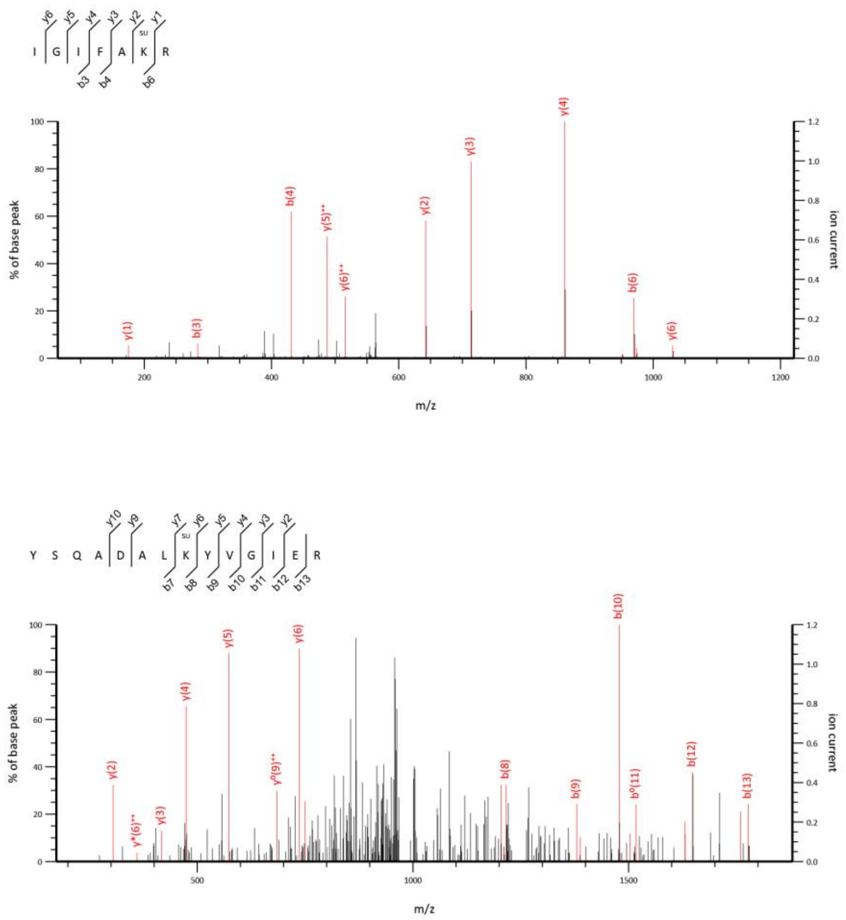

b

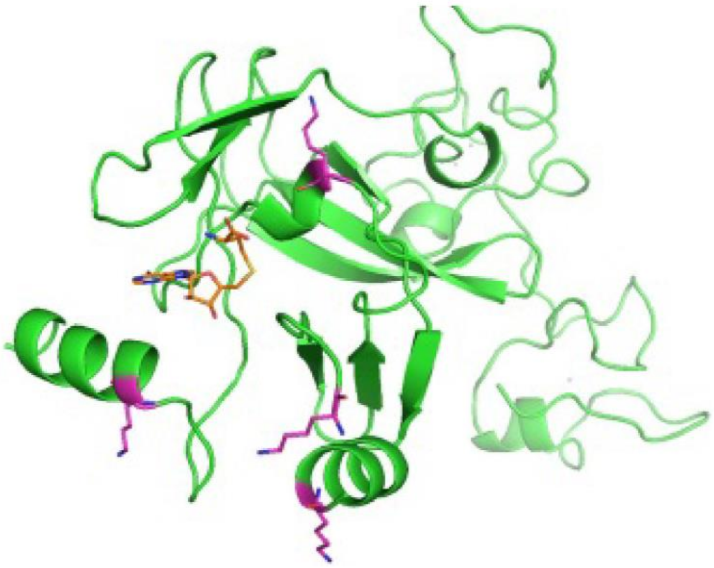

Figure S1. Generation of biotinylated PRC2 complexes.

a) MS/MS spectra of the biotinylated peptides. The upper panel shows peptides with the amino acid sequence IGIFAKR and the precursor mass [M+2H]2+ 572.34 Th and the lower panel shows peptides with the amino acid sequence YSQADALKYVGIER and the precursor mass [M+2H]2+ 976.50 Th. Matching fragment masses with indicated fragment ion series types are shown in red.
b) Representation of the sulfo-NHS-LC-biotin modified lysines (marked in magenta) at position 713 and 735 in the SET domain 3D structure of SAH-bound EZH2 (PDB: 5HYN)[13]. The EZH2 is colored in green, and SAH in orange.

Figure S2. Chemical structures and names of the reference compounds used for assay validation.

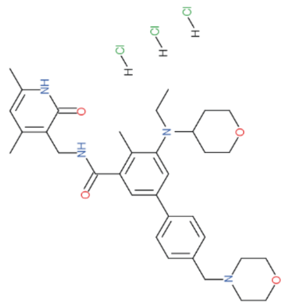

3: EPZ-6438 (3xHCl)

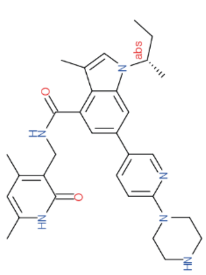

4: GSK126a

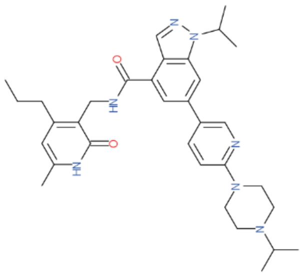

9: UNC1999

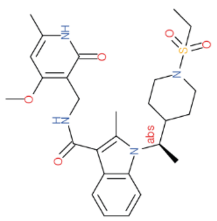

10: CPI-169

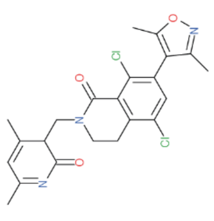

14: Pfizer compound 31

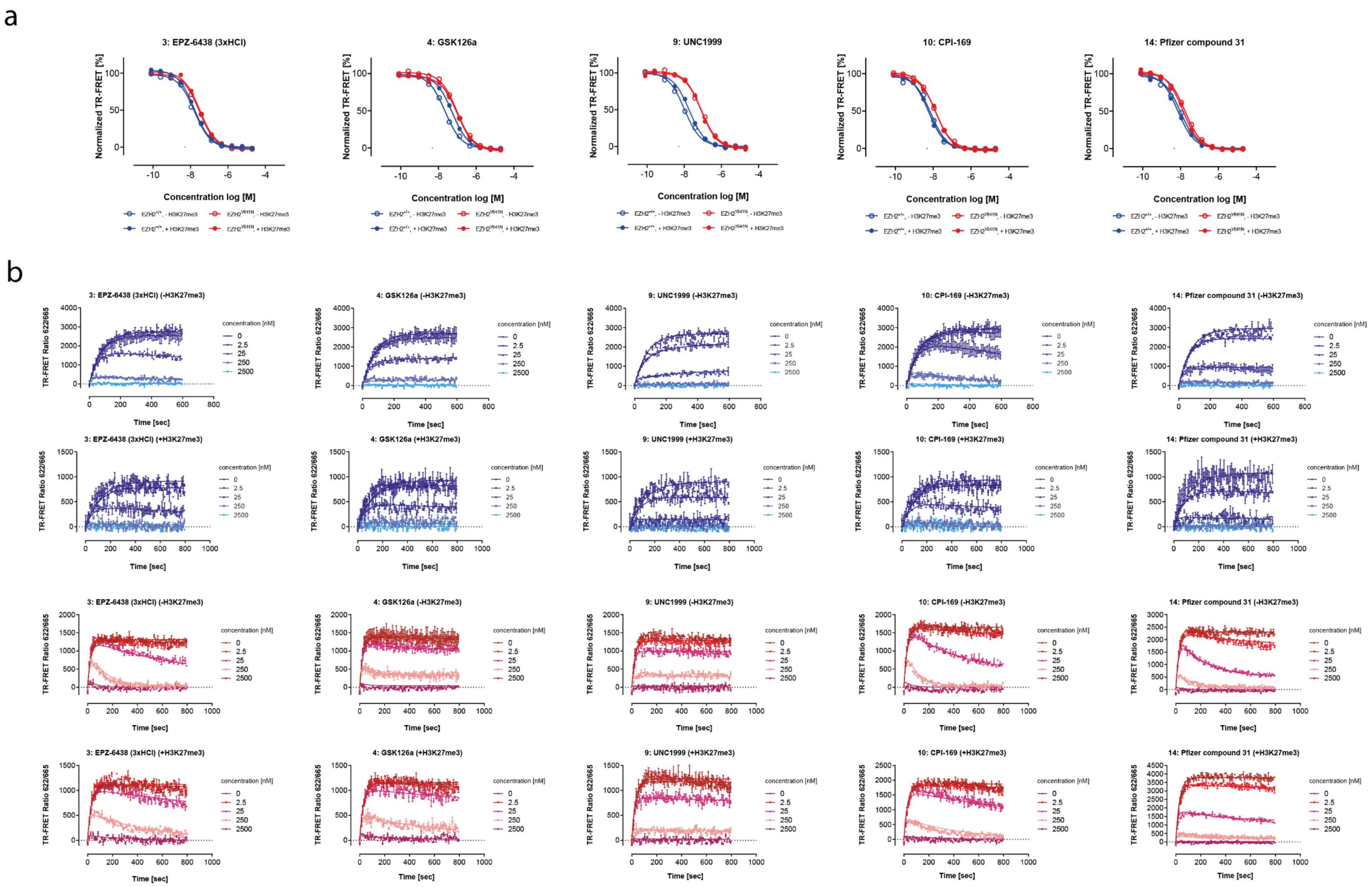

a
3: EPZ-6438 (3xHCl)
4: GSK126a
9: UNC1999
10: CPI-169
14: Pfizer compound 31
Normalized TR-FRET [%]
Concentration log [M]
EZH2+/+, - H3K27me3
EZH2+/+, + H3K27me3
EZH2Y641N, - H3K27me3
EZH2Y641N, + H3K27me3
b
3: EPZ-6438 (3xHCl) (-H3K27me3)
4: GSK126a (-H3K27me3)
9: UNC1999 (-H3K27me3)
10: CPI-169 (-H3K27me3)
14: Pfizer compound 31 (-H3K27me3)
3: EPZ-6438 (3xHCl) (+H3K27me3)
4: GSK126a (+H3K27me3)
9: UNC1999 (+H3K27me3)
10: CPI-169 (+H3K27me3)
14: Pfizer compound 31 (+H3K27me3)
TR-FRET Ratio 622/665
Time [sec]
concentration [nM]
0
2.5
25
250
2500

Figure S3. Representative equilibrium and kinetic TR-FRET assay data for reference compounds with wild type (blue traces) and Y641N mutant EZH2 (red traces).

a) Normalized steady state curves of fluorescent probe displacement from wild type and mutant EZH2 by reference inhibitors. Open and filled circles show competition in the absence and presence of saturating concentrations of the H3K27me3 peptide. Fitting these curves to a 4-parameter logistic model (solid lines), and conversion of the resulting IC_{50} values to Ki values with the Cheng-Prusoff equation, led to affinity parameters shown in the Supplementary Spreadsheet.
b) Representative kinetic probe competition assay (kPCA) traces for the same compounds in the absence and presence of saturating concentrations of the H3K27me3 peptide. Fitting these curves to the Motulsky and Mahan model for competitive binding kinetics (solid lines) yielded the kinetic parameters shown in the Supplementary Spreadsheet.

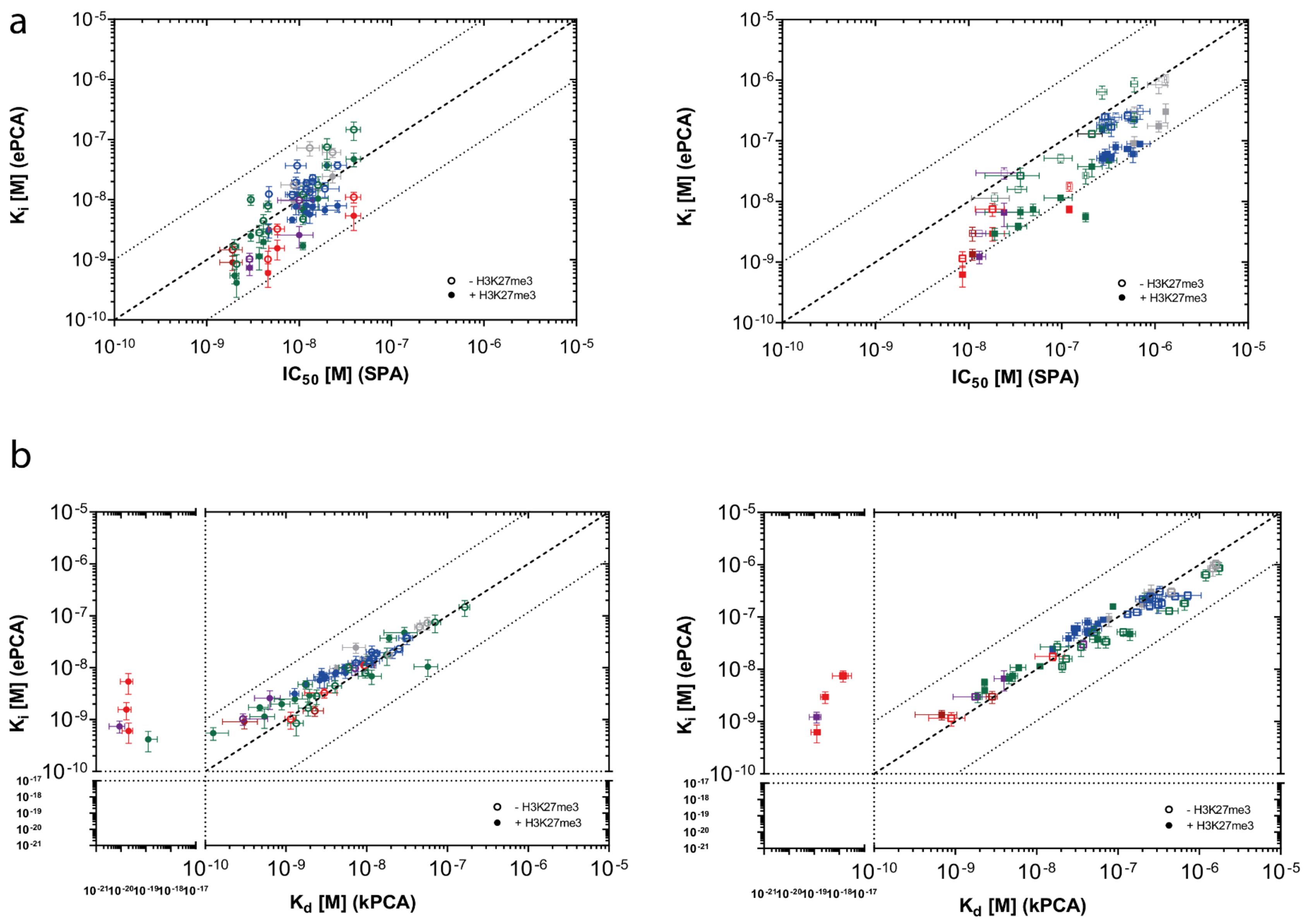
a
b
Ki [M] (ePCA)
IC50 [M] (SPA)
Kd [M] (kPCA)
- H3K27me3
+ H3K27me3

Figure S4. Validation of equilibrium and kinetic TR-FRET assays for wild type (left panels) and Y641N mutant EZH2 (right panels).

a) Comparison of inhibitory activity (IC_{50}) in enzymatic assays (x-axis) vs. equilibrium affinities from ePCA (y-axis).
b) Comparison of the affinities derived from kPCA) (x-axis) vs. ePCA (y-axis). Correlation outliers are compounds for which the off rates were not accurately determined in time allocated for kPCA to proceed. For these compounds the k_{off} values were calculated based on the k_{on} and ePCA Ki values (see main text).

For all sections the colors of the data points correspond to the scaffold names indicated in Figure 2A. The error bars represent standard deviations of at least 4 replicates in at least two independent experiments.

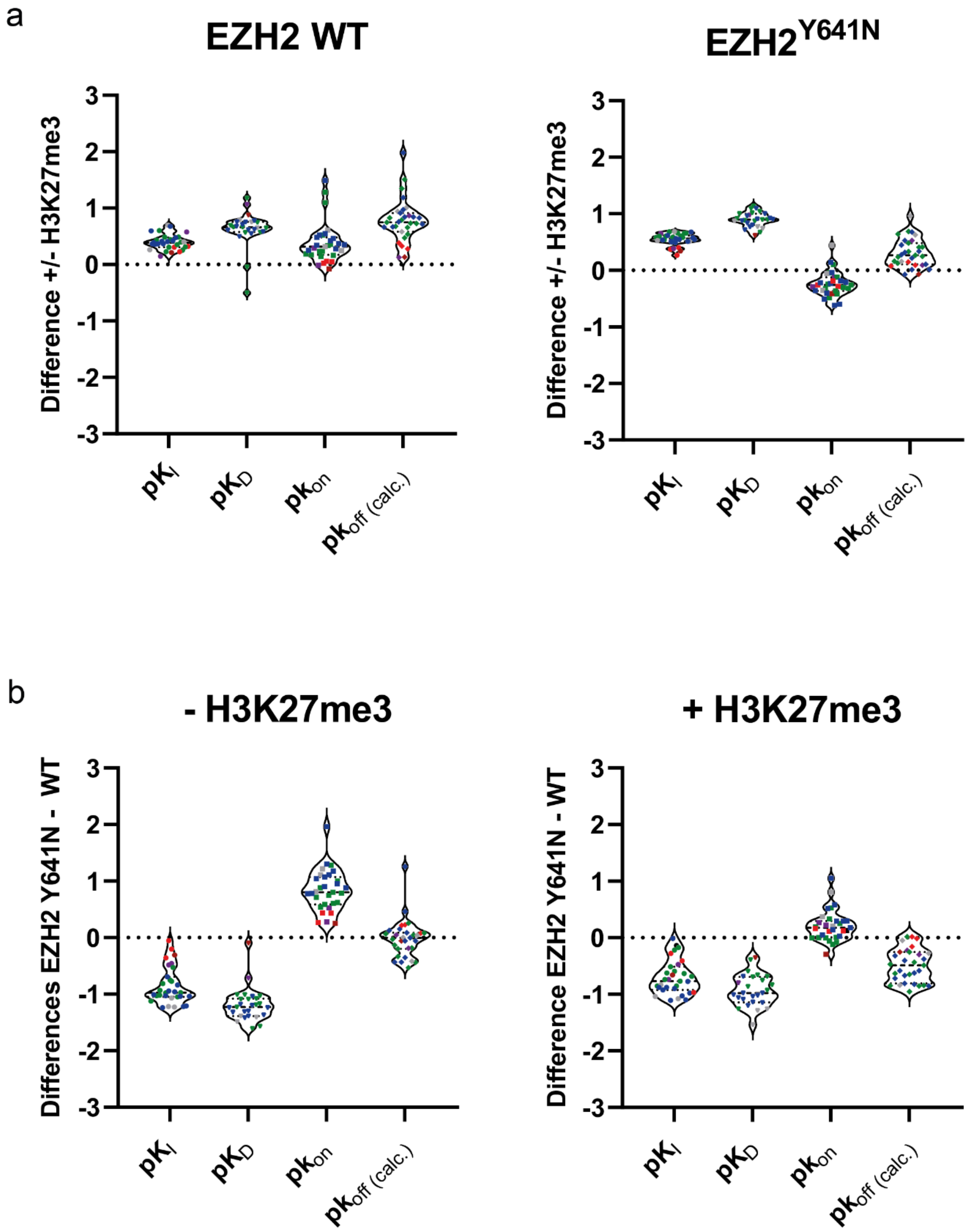

Figure S5. Violin plots representing the effects of H3K27me3 allosteric peptide on the affinity and kinetic parameters of inhibitor binding to wild type and Y641N mutant EZH2.

a) Changes in affinity and rate constants +/- treatment with the H3K27me3 allosteric peptide for $EZH2^{+/+}$ (left panel) and $EZH2^{Y641N}$ (right panel).
b) Changes in affinity and rate constants for binding to $EZH2^{Y641N}$ compared to $EZH2^{+/+}$ binding with and without treatment with the H3K27me3 allosteric peptide.

For all sections the colors of the data points correspond to the scaffold names indicated in Figure 2A.

.

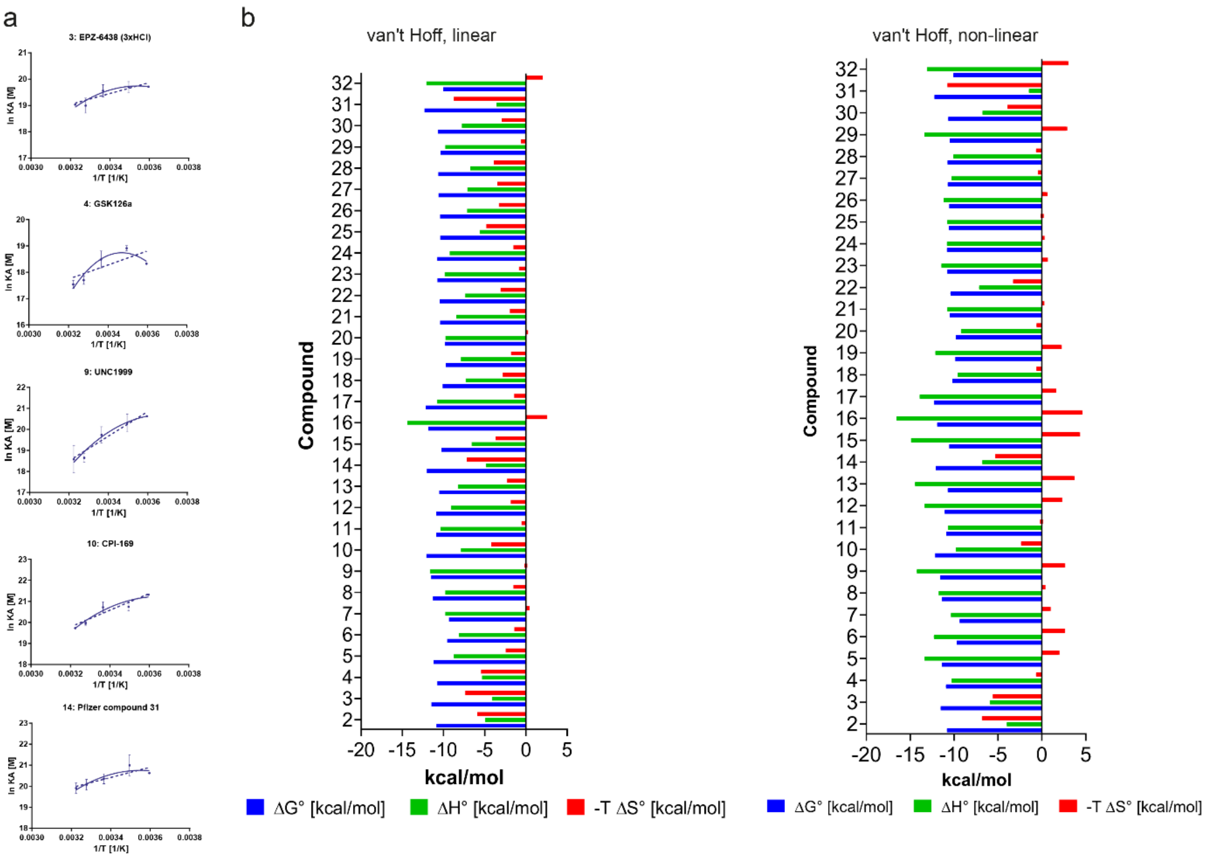

Figure S6. Equilibrium thermodynamics profiles of all compounds investigated in this study.

a) Equilibrium thermodynamics analysis derived from ePCA experiments for reference EZH2 inhibitors investigated in this study. Dotted lines represent the fit of the data to the linear form of the van't Hoff equation, whereas solid lines show the fit to the polynomial one.

b) Equilibrium thermodynamics profiles of all EZH2 inhibitors investigated in this study, calculated from ePCA experiments and analyzed with the linear polynomial van't Hoff equations (left and right panels, respectively). The blue, green and red bars represent the Gibbs' free energy (ΔG), enthalpy (ΔH) and entropy (-TΔS) components of the binding.

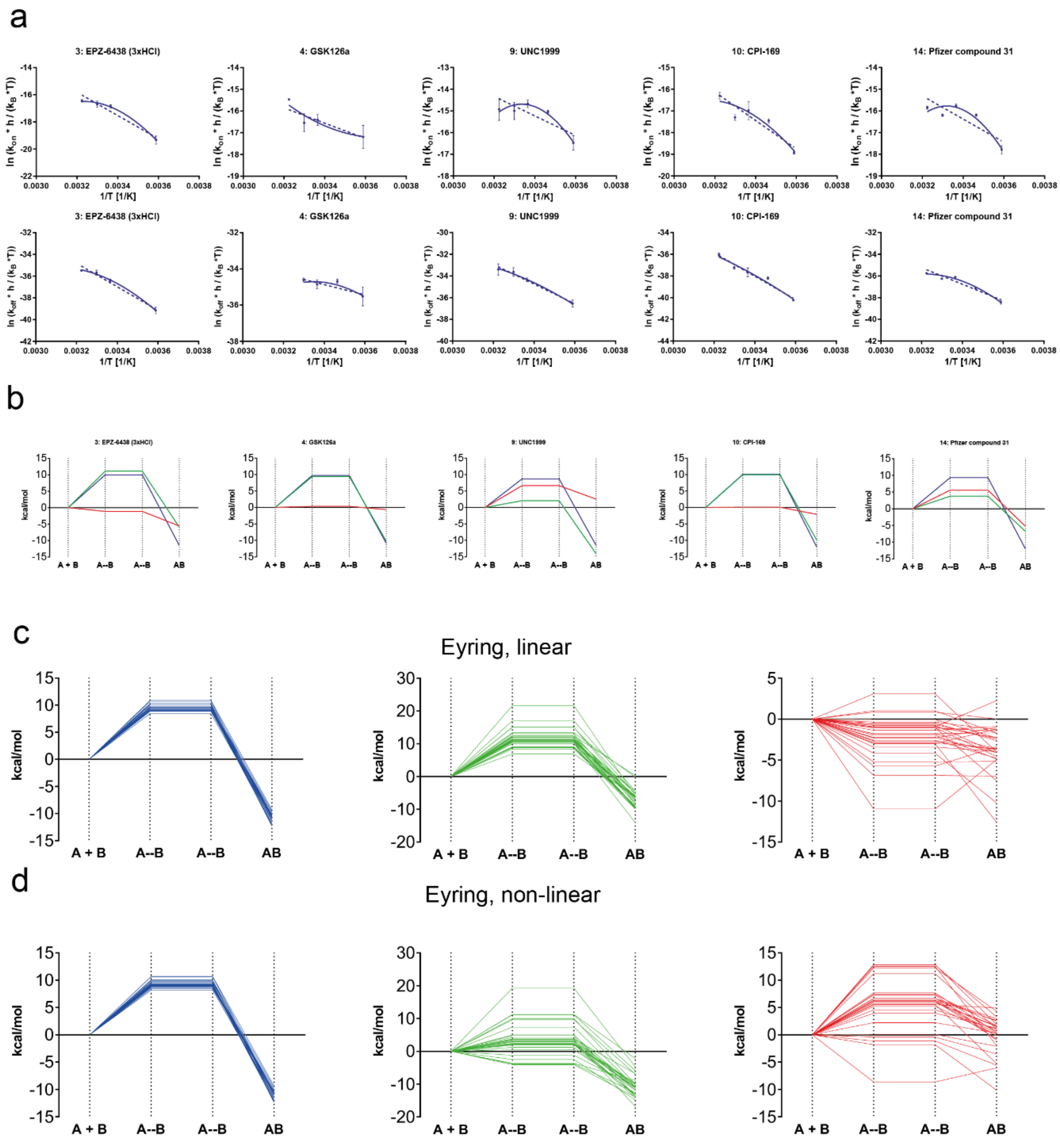

Figure S7. Transition state thermodynamics profiles of all compounds investigated in this study.

a) Transition state thermodynamics analysis derived from kPCA experiments for reference EZH2 inhibitors investigated in this study. Dotted lines represent the fit of the data to the linear form of the Eyring equation, whereas solid lines show the fit to the polynomial one. The upper panel shows the analysis for the association phase, whereas the bottom graphs display the dissociation phase.

b) Transition state thermodynamics profiles of reference EZH2 inhibitors investigated in this study. The blue, green and red bars represent the Gibbs' free energy (ΔG) enthalpy (ΔH), and entropy (-TΔS) components of the binding, respectively.

c) Transition state thermodynamics profiles of all EZH2 inhibitors investigated in this study, calculated from kPCA experiments and analyzed with the linear Eyring equation. The

blue, green and red bars represent the Gibbs' free energy (ΔG), enthalpy (ΔH) and entropy (-TΔS) components of the binding, respectively.

d) Transition state thermodynamics profiles of all EZH2 inhibitors investigated in this study, calculated from kPCA experiments and analyzed with the polynomial Eyring equation. The blue, green, and red bars represent the Gibbs' free energy (ΔG), enthalpy (ΔH) and entropy (-TΔS) components of the binding, respectively.

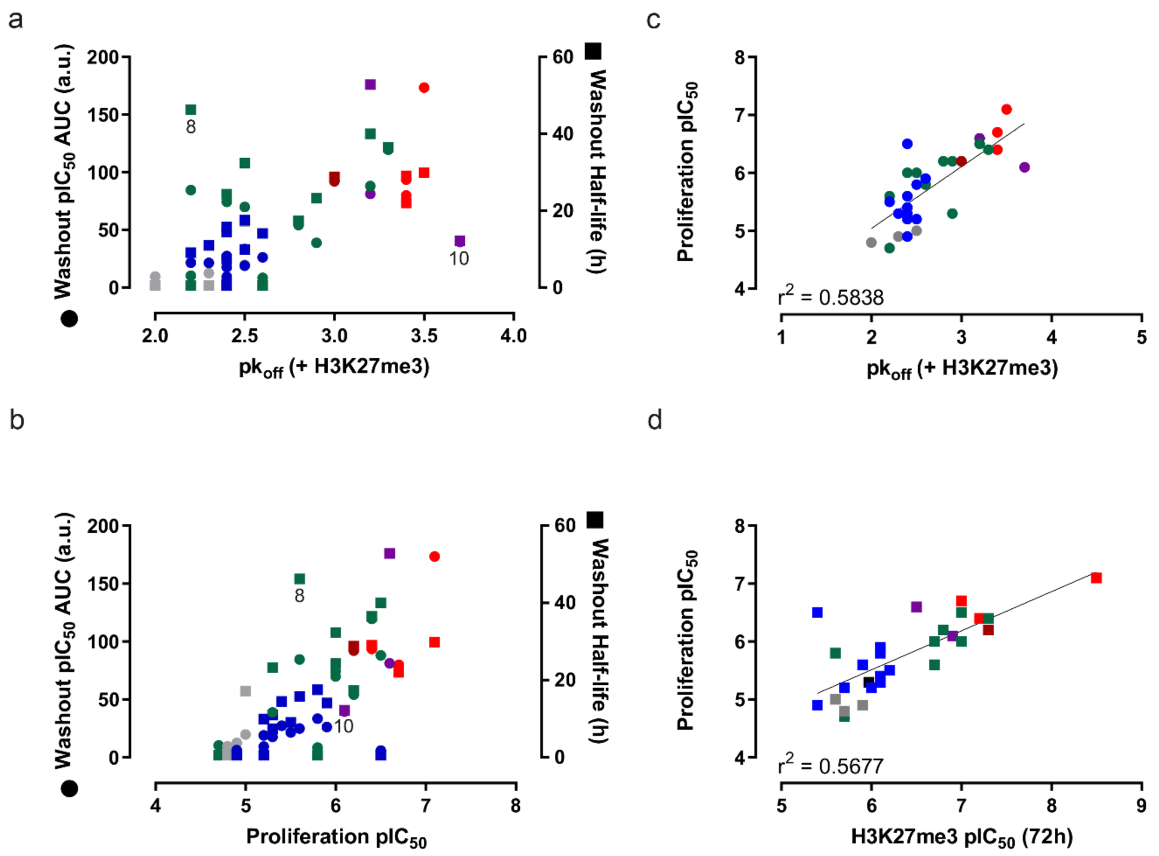

Figure S8. Comparison of target dissociation rate- and cellular target engagement-related parameters with KARPAS anti-proliferative activity.

a) Scatter plot of pk_{off} values from kPCA binding assays run in presence of saturating concentrations of the H3K27me3 peptide (x-axis) vs. HCA H3K27 methylation washout pIC_{50} AUC (left y-axis) and half-lifes (right y-axis).
b) Scatter plot of KARPAS anti-proliferation pIC_{50} values (x-axis) vs. HCA H3K27 methylation washout pIC_{50} AUC (left y-axis) and half-lifes (right y-axis).
c) Scatter plot of pk_{off} values from kPCA binding assays (x-axis) run in presence of saturating concentrations of the H3K27me3 peptide vs. antiproliferation pIC_{50}.
d) Scatter plot of pIC50 values from 72-hour HCA H3K27 methylation assay (x-axis) vs. antiproliferation pIC_{50}.

For all sections the colors of the data points correspond to the scaffold names indicated in Figure 2A. Compounds 8 and 10 (CPI-169) discussed in the main text as extreme outliers are labeled in both panels.

Supplementary Tables

Supplementary Table S1. Binding and kinetic parameters of the fluorescent EZH2 probe. AP: activator peptide (H3K27me3).

Protein	*AP*	*Temp. (°C)*	*Equilibrium*			*Kinetic*		
			**K_D [M]**	**AP K_b [M]**	**AP Alpha**	**k_{on} [s^{-1} M^{-1}]**	**k_{off} [s^{-1}]**	**K_D [M]**
EZH2 +/+	-	*25*	1.13E-09 ± 1.83E-10	-	-	1.96E+06 ± 3.64E+05	2.73E-03 ± 9.20E-05	1.433E-09 ± 2.19E-10
	+		3.93E-10 ± 8.80E-12	2.11E-06 ± 7.67E-07	2.86 ± 0.37	1.16E+06 ± 2.30E+05	7.28E-04 ± 4.14E-05	6.53E-10 ± 1.43E-10
	-	*5*	-	-	-	1.52E+05 ± 1.82E+03	1.77E-04 ± 1.38E-06	1.16E-09 ± 1.83E-11
	-	*15*	-	-	-	9.53E+05 ± 8.23E+03	5.05E-04 ± 2.86E-06	5.30E-10 ± 5.77E-12
	-	*30*	-	-	-	8.53E+05 ± 1.54E+04	5.10E-03 ± 7.49E-05	5.98E-09 ± 9.30E-11
	-	*37*	-	-	-	2.22E+06 ± 3.67E+04	8.23E-03 ± 1.16E-04	3.71E-09 ± 4.88E-11
EZH2 Y641N	-	*25*	9.56E-09 ± 3.40E-09	-	-	6.79E+05 ± 1.51E+05	1.77E-02 ± 1.05E-03	2.70E-08 ± 4.48E-09
	+		2.72E-09 ± 4.69E-10	5.52E-07 ± 1.08E-07	6.05 ± 2.82	1.01E+06 ± 3.38E+05	3.45E-03 ± 2.89E-04	3.76E-09 ± 9.73E-10
